# RHOAming Through the Nucleotide Excision Repair Pathway as a Mechanism of Cellular Response Against the Effects of UV Radiation

**DOI:** 10.3389/fcell.2020.00816

**Published:** 2020-08-19

**Authors:** Yuli T. Magalhaes, Gisele E. T. Silva, Juliana H. Osaki, Clarissa R. R. Rocha, Fabio L. Forti

**Affiliations:** ^1^Biomolecular Systems Signaling Laboratory, Department of Biochemistry, Institute of Chemistry, University of São Paulo, São Paulo, Brazil; ^2^DNA Repair Laboratory, Department of Microbiology, Biomedical Sciences Institute, University of São Paulo, São Paulo, Brazil

**Keywords:** Rho GTPases, UV radiation, DNA damage response pathway, nucleotide excision repair pathway, cell cycle and proliferation

## Abstract

Typical Rho GTPases include the enzymes RhoA, Rac1, and Cdc42 that act as molecular switches to regulate essential cellular processes in eukaryotic cells such as actomyosin dynamics, cell cycle, adhesion, death and differentiation. Recently, it has been shown that different conditions modulate the activity of these enzymes, but their functions still need to be better understood. Here we examine the interplay between RhoA and the NER (Nucleotide Excision Repair) pathway in human cells exposed to UVA, UVB or UVC radiation. The results show high levels and accumulation of UV-induced DNA lesions (strand breaks and cyclobutane pyrimidine dimers, CPDs) in different cells with RhoA loss of function (*LoF*), either by stable overexpression of negative dominant RhoA (RhoA-N19 mutant), by inhibition with C3 toxin or by transient silencing with siRNA. Cells under RhoA *LoF* showed reduced levels of γH2AX, p-Chk1 (Ser345) and p-p53 (Ser15) that reflected causally in their accumulation in G1/S phases, in low survival rates and in reduced cell proliferation, also in accordance with the energy of applied UV light. Even NER-deficient cells (XPA, XPC) or DNA translesion synthesis (TLS)-deficient cells (XPV) showed substantial hypersensitivity to UV effects when previously submitted to RhoA *LoF*. In contrast, analyses of apoptosis, necrosis, autophagy and senescence revealed that all cells displaying normal levels of active RhoA (RhoA-GTP) are more resistant to UV-promoted cell death. This work reaffirms the role of RhoA protein signaling in protecting cells from damage caused by UV radiation and demonstrates relevant communicating mechanisms between actin cytoskeleton and genomic stability.

## Introduction

The maintenance of genomic stability is essential to cellular physiology and survival, and many diseases occur due to disturbances in that process. In-depth study of the intrinsic pathways that regulate genomic stability is essential for the knowledge and development of new therapeutic methods. Emerging as new and important regulatory components for maintaining genomic stability are the typical Rho GTPases, a small family of signaling molecules described as important regulators of cell and tissue morphology and function, acting mainly through the actin cytoskeleton. These enzymes are key mediators of diverse cellular and physiological processes such as cell division, migration and invasion (Mokady and Meiri, 2015; Al-Koussa et al., 2020). RhoA, RhoB, and RhoC isoforms comprise the Rho subfamily within the Rho GTPase family. These three proteins have a high degree of sequence similarity, although presenting in some specific cellular contexts, very distinct roles. RhoA, RhoB and RhoC proteins are often expressed aberrantly in human tumors, with RhoA and RhoC being overexpressed, whereas RhoB is usually downregulated (Tseliou et al., 2016). RhoA and RhoC are often described as oncogenes, acting on cell survival under DNA damage, while RhoB is frequently recognized as a tumor suppressor. However, under different types of DNA damaging agents, these enzymes present high activity and expression, acting as pro-survival factors and implicated in the regulation of components of DNA damage response pathways. However, only few details about the mechanisms underlying these processes occurrence are known (Dubash et al., 2011; Mamouni et al., 2014; Fritz and Henninger, 2015; Espinha et al., 2016; Herraiz et al., 2016).

UV radiation is part of the spectrum of electromagnetic radiation emitted by the sun and excessive exposure to it can be seriously harmful for biomolecules such as proteins, lipids, RNA, and especially DNA. The direct exposure to UV generates dangerous lesions to DNA, such as CPD (cyclobutane pyrimidine dimers) and 6-4PPs (6-4 pyrimidine-pyrimidone photoproducts). UV can also lead to lesions induced by oxidation, mostly selective guanine oxidation that produces primarily 8-oxo-7,8-dihydroguanine (8-oxoG). UVA is the most responsible for oxidative bases and single strand breaks, while UVB and UVC are the most responsible for photoproducts (Schuch et al., 2017; Emri et al., 2018). DNA photoproducts, especially CPDs, are the major pre-mutagenic and genotoxic lesions, leading to higher level of mutagenesis, cell cycle arrest and cell death (Rünger and Kappes, 2008; Schuch and Menck, 2010; Schuch et al., 2017; Mullenders, 2018). To avoid extensive cell death under solar exposition, cells count with sophisticate DNA damage response and repair mechanisms.

In the context of UV-damaged DNA, the major pathway activated is the Nucleotide Excision Repair (NER). The NER can be initiated by two distinct recognition mechanisms: transcription-coupled repair (TCR), which detects and removes damage from active genes in transcription, and global genome repair (GGR), which removes UV-induced damage present across the genome. The TCR is activated when a bulky adduct blocks the action of the RNA polymerase II (RNAPII). In this case, the attached elongation complex recruits CSB (ERCC6), which in turn binds strongly to RNA polymerase and alters DNA conformation, changing the interface between RNAPII and DNA. The CSB recruits the CSA complex, the NER factors (not including GGR factors XPC and XPE) and p300 at the linked RNAPII sites. The polymerase is then removed to allow access to TFIIH and other NER repair enzymes to the lesion site (Spivak, 2015). In GGR, XPC complexed with RAD23B and centrin 2 (CETN2) directly recognizes the lesion distorting the DNA helix. For CPD lesions, which do not significantly destabilize the duplexes, the lesion is firstly recognized by XPE (DDB2) in complex with DDB1 (Spivak, 2015). This creates a greater distortion that is recognized by XPC, which has the capacity of recognizing diverse types of lesions, not necessarily repaired by NER, due to its ability to bind the strand opposite to the lesion (Lee et al., 2014; Spivak, 2015). The XPC-RAD23b-CETN2 complex erases the DNA around the lesion and recruits the multiprotein complex TFIIH. Both TCR and GGR converge on a single path, recruiting the NER system, including the general transcription factor TFIIH, XPA and the endonucleases XPF and XPG. The lack of NER genes and dysfunction of the pathway causes a syndrome called Xeroderma Pigmentosum (XP), that leads to a higher sensitivity to UV-light and increased susceptibility to skin cancer (Oh et al., 2011).

Another pathway involved in repairing UV-induced DNA damage tolerance is the translesion synthesis (TLS) pathway. In this mechanism, specialized DNA polymerases act as a bypass system, being recruited to damaged DNA sites and promoting replication across the lesion. This process is highly error-prone and is the major source of DNA damage-induced mutagenesis (Zhao and Todd Washington, 2017). However, DNA polymerase eta (Polη) suppresses efficiently the induction of mutations after UV radiation by performing an error-free TLS using the base-pairing ability, even if the CPD lesion still remains. Patients of a variant form of Xeroderma Pigmentosum (XPV), which have deficiencies in the *POLH* gene, show high photocarcinogenic sensitivity in skin regions exposed to sunlight, and cells removed from such patients are also sensitive to UV-induced mutations (Ikehata and Ono, 2011).

UV-induced DNA breaks can occur in two different (but simultaneously) situations: due to UV radiation by itself or due some failure during the repair processing. UV radiation photons can primarily break chemical bonds, especially the high energy ones, leading to small amounts of single or double strand breaks (S/DSB) not very often observed. UV radiation also can lead to secondary DNA breaks, where the typical UV-induced lesions, such as CPD and 6-4PP, accumulate in the DNA, generating high tension in the DNA helix (which can lead to breaks) or mainly blocking the replication and/or transcription mechanisms (and also generating replicative stress caused by the base mismatch due to oxidative lesions) (Rastogi et al., 2010). During NER functioning the DNA is resected to promote the excision of the damage region and every single time NER is not correctly performed or stopped at some step, it can cause the production of DSBs (Wakasugi et al., 2014).

The NER pathway activation is a process also linked to the DNA damage response (DDR) pathway. Under DNA damage, G1/S and G2/M checkpoints of the cell cycle are activated. Checkpoint activation is mainly controlled by two kinases belonging to the PIKK superfamily, the ataxia telangiectasia mutated (ATM) and the ataxia telangiectasia and Rad3 related (ATR). ATR kinase is a primary key regulator of the NER pathway able to detect the DNA stress caused by UV-induced damage. During NER mechanism ATR, in complex with its nuclear binding partner ATR-interacting protein (ATRIP), binds to RPA-coated ssDNA generated by XPF/ERCC1 endonuclease complex and Exo1 activity, leading to the DDR signaling and cell cycle arrest through the Chk1 activation (Sertic et al., 2012; Musich et al., 2017). XPA protein accumulates in the nucleus after UV-exposure in a ATR-dependent manner, but not ATM (Wu et al., 2007), but, despite this information about DDR – NER mechanisms, many regulatory processes involved in the cellular responses are still unknown.

In this work, we show some roles of Rho GTPase enzymes in protecting cells from damage caused by UV radiation and identified which isoform of these enzymes are best regulators of the NER and/or DDR pathways, demonstrating an underestimated interplay and dependency between actin cytoskeleton and genomic stability.

## Materials and Methods

### Cell Lines and Culture Conditions

HeLa cells (Espinha et al., 2015), MRC-5V1 (MRC5) fibroblasts, XP12RO (XPA) and XP4PA (XPC) NER-deficient cell lines, and XP30RO (XPV) TLS-deficient cell line (de Lima-Bessa et al., 2008) were cultured in DMEM with 10% FBS, 25 μg/mL ampicillin and 100 μg/mL streptomycin at 37°C and 5% CO_2_. The dominant negative HeLa RhoA-N19 (Thr to Asp substitution at position 19) and the constitutively active HeLa-RhoA-V14 (Gly to Val substitution at position 14) were generated and characterized previously (Osaki et al., 2016) and cultured in DMEM with 100 μg/mL G418.

### Rho LoF by C3 Toxin Treatment and RhoA/RhoB Knockdown Using siRNA

The inhibition of Rho activity or Rho loss of function (*LoF*) was performed by transient transfection of the eukaryotic expression vector pEF-myc containing the C3 toxin coding sequence (Osaki et al., 2016). Cells were transfected using Lipofectamine 3000 (Invitrogen) for 24 h, according to the manufacturer’s instructions. For gene silencing, HeLa cells were transfected with specific siRNAs for RhoA or RhoB genes (MISSION^®^ esiRNA, Invitrogen) and Lipofectamine 3000.

### Ultraviolet Radiation Treatments

For UV treatments, culture medium was removed, and cells were exposed to one out of the three different and specific UV wavelengths (365 nm for UVA, 302 nm for UVB and 260 nm for UVC) for the appropriated time needed to reach the desired doses. The VLX-3W dosimeter (Vilber Lourmat, Germany), coupled with specific probes for each wavelength, was used to determine the exposure times and for keeping the lamps calibrated.

### Cellular Growth Curves

Cells were plated in a density of 3.5–5 × 10^4^ cells per 35 mm diameter dish plate, and treated 24 h later accordingly. After the treatments, cells were trypsinized and fixed in 10% formaldehyde in PBS, every 24 h for five consecutive days, and finally counted in a Fuchs-Rosenthal chamber. The data were showed as total number of cells daily counted.

### Clonogenic Survival Assays

For these experiments using C3 toxin or siRNAs, cells were previously transfected according to the appropriated time. In monolayer colony assays, isolated colonies were obtained from plating cells at low density (1 × 10^4^ for HeLa and MRC5 fibroblasts, and 2 × 10^4^ for NER- and TLS-deficient cells). Cells were irradiated 24 h later and allowed to growth for 10–12 days with medium replacement every 3 days, then were fixed with 10% formaldehyde and stained with 0.5% crystal violet. Colonies containing more than 50 cells were counted. The soft agar assays were performed as previously describe (Borowicz et al., 2014) with modifications. Briefly, 1.5 × 10^3^ cells previously treated and irradiated were resuspended in 1 mL of culture medium containing 0.3% agarose. This suspension was added onto a solidified layer of medium containing 0.6% agarose in 24-well plates. 500 μL of medium was added onto the agarose matrix and replaced every three days. Colonies were allowed to grow for 3–4 weeks and subsequently stained with 0.01% crystal violet in 70% ethanol. The wells were photographed and quantified by Image J software, through the plugin Cell Counter. All survival data were presented as survival fraction (%), where the control condition without radiation and without Rho inhibition or knockdown being assumed as 100% survival. The fold change was taken by the ratio between the irradiated cells and not irradiated for each group (Control cells, + C3, + siRNA, and RhoA-N19 mutants).

### Cell Cycle Analysis by Flow Cytometry

HeLa cells and RhoA-N19 clones were exposed to UV-radiation and collected, fixed in 70% cold ethanol, centrifuged at 1500 rpm for 5 min and stored at 4°C until the day of analysis. The samples were stained with 2μg/mL propidium iodide containing 0.1% Triton X-100, 0.1% sodium citrate and 10 μg/mL RNAse for 20 min at room temperature. For analysis, 30,000 events of each sample were read in a FACS Verse Flow Cytometer (BD Biosciences) and data was analyzed using Kaluza^®^ 1.3 Analysis software (Beckman Coulter). For data representation, the percentage of cells distribution in each cell cycle phase was plotted, where the sum of all phases was assumed as 100%.

### Senescence-Associated β-Galactosidase Assays

HeLa and RhoA-N19 cells were exposed to UV-treatments. After 96 h, cells were fixed with 2% formaldehyde and 0.2% glutaraldehyde in PBS for 3min and stained for 18h at 37°C with 2mL of X-gal staining solution (30mmol/L citric acid, 5mmol/L K_3_Fe(CN)_6_, 2mmol/L MgCl_2_, 150mmol/L NaCl, 5mmol/L K_4_Fe(CN)_6_, and 1mg/mL X-gal, in PBS pH 6,0). Then, samples were washed twice with PBS and kept at 4°C. The analysis was made by direct counting of β-galactosidase-positive/negative cells (at least 1 × 10^3^ cells per sample), in an inverted Olympus microscope (Olympus, Tokyo, Japan). The data were presented as percentage of senescent (blue stained) cells.

### Cell Death Analysis by Flow Cytometry Using Annexin-V/Propidium Iodide Staining

To estimate different apoptosis phases (early and late) and necrosis, HeLa and RhoA-N19 cells were treated with UV-radiation and collected (including the supernatants possibly containing cells) 48h and 72h after stress. Cells were then resuspended in Annexin-V binding buffer (50mM HEPES, pH 7.4, containing 0.7M NaCl and 12.5mM CaCl_2_) in a final density of 1 × 10^6^ cells/mL. In aliquots of 100 μL of cell suspension (containing 1 × 10^5^ cells) were added 5μL of Annexin-V-FITC (BD Biosciences) and 1.5μL of 1 mg/mL propidium iodide. Samples were incubated for 15min at room temperature in a dark chamber and 400μL of Annexin-V binding buffer was added to each sample. Cells were analyzed by flow cytometry in a FACS Verse (BD Biosciences) and the data were analyzed on the Kaluza^®^ 1.3 Flow Analysis software (Beckman Coulter). The results were presented as percentage of cells in early apoptosis (positive for Annexin V and negative for PI), late apoptosis (positive for both Annexin V an PI) and necrosis (negative for Annexin V and positive for PI).

### Alkaline Comet Assays

Alkaline comet assay was performed as described (Magalhães et al., 2018). Briefly, cells were exposed to UV and collected at different timepoints. Cells were mixed with 0.5% low melting-point agarose and applied onto a glass slide covered with a thin layer of 1.5% agarose. Then cells were lysed and submitted to electrophoresis at constant voltage of 25 V for 30 min. The slides were neutralized with 0.4 M Tris-HCl pH 7.5, fixed with ethanol and stained with 2 μg/mL ethidium bromide. 100 nuclei from each slide were photographed in a fluorescence microscope (Olympus BX51). DNA fragmentation was expressed as the Olive Tail Moment (OTM) parameter by using the Komet 6.0 software (Andor Technology). Data in form of bars graph were also submitted to a linear regression analyses using the post-irradiation time-points to estimate the repair rate, where the repair speed was considered proportional to the slope.

### Host Cell Reactivation (HCR) Assays

The HCR assay was performed as described previously (Russo et al., 2018). The plasmids carrying the reporter genes (pShuttle MCS for luciferase and pRL SV40 for renilla) were previously treated with different doses and wavelengths of UV-radiation to generate DNA lesions. 2 × 10^4^ cells were plated in 96-well plates and transfected with the UV-damaged plasmids. The repair of UV-promoted lesions was associated with reactivation of luciferase expression by the Dual-Glo Luciferase Assay Systems kit (Promega), where the luminescence was detected in a GloMax^®^ luminometer (Promega). The luminescence associated to the plasmid without radiation treatment was considered as 100% of repair.

### Detection of Cyclobutane Pyrimidine Dimers (CPD)

For the detection of CPD lesions by slot-blot assays (Russo et al., 2018) genomic DNA was extracted after UV-radiation. Hundred nanogram of each sample was denatured and transferred to a nitrocellulose membrane through vacuum. The membrane was fixed at 80°C, blocked with 5% non-fat milk for 18 h at 4°C, incubated with the primary anti-CPD (Table 1) and secondary antibodies, scanned using an Odyssey infrared imaging system (Li-Cor) and quantified using the Image Studio software (Li-Cor). By using immunofluorescence for CPD detection, cells were fixed with 4% PFA for 5 min and permeabilized with 0.5% Triton X-100 for 5 min on ice. The DNA was denatured with 2 M HCl for 30 min at 90°C and cells were blocked with 3% BSA/10% FSB for 30 min and incubated with the anti-CPD antibody (1:200 in PBS) for 2 h at 4°C following incubation with a secondary antibody anti-mouse Alexa Fluor 568 (Invitrogen) for 1 h. Images acquisition was done with a Zeiss LSM-510 microscope. The Image Studio software was used to obtain the densitometry of each immuno slot-blot bands. The normalization was carried out assuming the not irradiated condition as 0% of CPD lesions and the first point after UV as 100% of CPDs, in each group (Control cells, + C3 and + siRNA). Data in form of bars graph were also submitted to a linear regression analyses using the post-irradiation time-points to estimate the repair rate, where the repair speed was considered proportional to the slope.

**TABLE 1:** Antibody features for Western blotting assays.

Antibody	Dilution	Incubation conditions	Source	Company	CAT #
Actin	1:1000	2 h, RT	Goat	Santa Cruz Biotechnology	sc-10731
p-Chk1 (Ser345)	1:1000	Overnight, 4°C	Rabbit	Cell Signaling Technology	2341
Chk1	1:500	Overnight, 4°C	Mouse	Cell Signaling Technology	2360
p53	1:1000	Overnight, 4°C	Mouse	Santa Cruz Biotechnology	sc-56180
p-p53 (Ser15)	1:1000	Overnight, 4°C	Rabbit	Cell Signaling Technology	9284
p-H2AX (Ser139)	1:1500	Overnight, 4°C	Rabbit	R&D Systems	AF2288
RhoA	1:500	4 h, RT	Mouse	Santa Cruz Biotechnology	sc-418
RhoB	1:500	4 h, RT	Rabbit	Santa Cruz Biotechnology	sc-180
CPD	1:500	2 h, RT	Mouse	Cosmo Bio Co.	–
Secondary IRDye	1:15,000	1 h, RT	–	LI-COR Biosciences	–

### Western Blottings

Cells were lysed with RIPA lysis buffer (50 mM Tris pH 7.2, 1% Triton X-100, 0.5% sodium deoxycholate, 0.1% SDS, 500 mM NaCl, 10 mM MgCl_2_, 1 mM Na_3_VO_4_, 1 mM NaF, 10 μg/mL each of aprotinin and leupeptin, and 1 mM PMSF). Proteins were quantified by Bradford colorimetric method and 100 μg were denatured with Laemmli buffer, resolved in SDS-PAGE and transferred to a nitrocellulose membrane (Millipore). The membrane was blocked with 5% low fat milk for 1 h and incubated with the specific primary and secondary antibodies (Table 1). Finally, the membranes were scanned using an Odyssey infrared imaging system and quantified using Image Studio software (LI-COR). The bands densitometry was performed with the software Image Studio. Each band density was obtained by the ratio of phosphorylated proteins and loading control, and the fold change was calculated by the ratio between each point after radiation and the not irradiated control.

### Statistical Analysis

Comparisons between treatments were performed by Two-way ANOVA with Tukey post-test, using the Prism 6.0 software, and differences were considered statistically significant when *p* < 0.05. The statistical was considered (^∗^) when 0.05 ≥ *p* > 0.001, (^∗∗^) when 0.01 ≥ *p* > 0.001, (^∗∗∗^) when 0.001 ≥ *p* ≥ 0.0001, and (^****^) when *p* < 0.0001. Statistical analysis was performed between control and RhoA *LoF* cells always at the same treatment conditions.

## Results

### Different Strategies Used for RhoA LoF in HeLa Cells Cause Strong Antiproliferative Effects When Combined With Different UV Wavelengths

RhoA loss of function (*LoF*) in HeLa cells was performed by three different molecular strategies: (i) direct inhibition, achieved through the transient transfection with the pEF-myc vector containing the C3 transferase from *Clostridium botulinum* bacteria, which is a toxin that specifically inhibits the three isoforms of Rho GTPase (RhoA/RhoB/RhoC) through the N-ADP ribosylation of the asparagine 41 residue at the GTPase binding site (Han et al., 2001; Vogelsgesang et al., 2007). The C3 toxin strongly affected cell morphology and actin filaments integrity (Supplementary Figures 1A–C); (ii) RhoA and RhoB knockdown, performed using specific siRNAs transiently transfected (Supplementary Figures 1D,E). RhoC knockdown was not performed since the parental HeLa cells did not express this GTPase (Supplementary Figure 1F); (iii) downregulation of endogenous RhoA activity, obtained by stably overexpressing the dominant negative RhoA-N19 mutant to generate the subline HeLa RhoA-N19, previously described as deficient in RhoA activity (Osaki et al., 2016).

**FIGURE 1:**
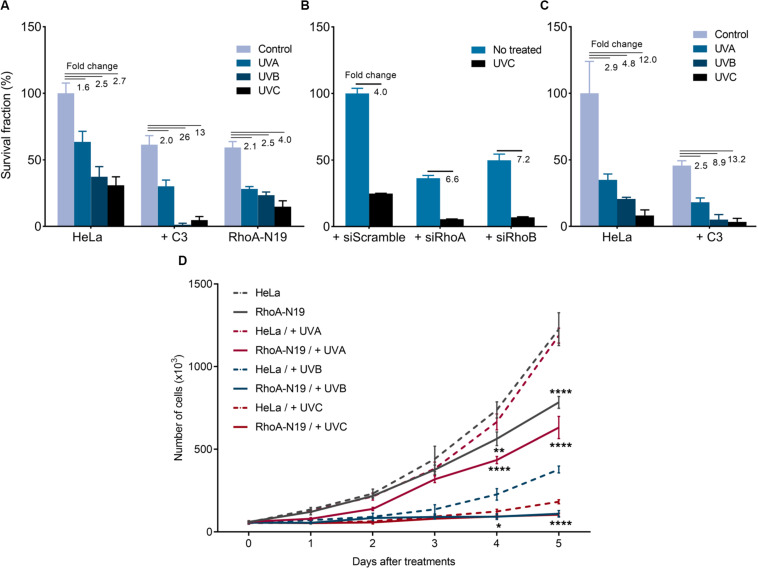
Rho inhibition previously to the UV radiation treatments further reduces survival and proliferation of HeLa cells. 2D-clonogenic assays of HeLa cells under Rho inhibition by the C3 toxin or overexpressing the dominant negative (N19) mutant **(A)** or submitted to knockdown of RhoA or RhoB by specific siRNAs **(B)**, and 3D-clonogenic assays in soft-agar of HeLa cells under Rho inhibition by C3 toxin **(C)** shows a reduced cell survival in response to UV stress that was enhanced by RhoA *LoF*. **(D)** Growth curves of HeLa cells and RhoA-N19 mutant clone show a decrease in proliferation after UV radiation, also enhanced by RhoA *LoF*. Graphs show mean ± SD of three independent experiments. Two-way ANOVA: (*) 0.01 ≤ *p* < 0.05, (**) 0.001 ≤ *p* < 0.01, and (****) *p* < 0.0001.

UV-light treatments reduced both survival and proliferation of HeLa cells, and this effect was enhanced by RhoA *LoF* (Figure 1). The UVA (50 kJ/m^2^), UVB (80 J/m^2^), and UVC (6 J/m^2^) irradiation decreased clonogenic survival of HeLa cells with a fold decrease of 1.6, 2.5, and 2.7, respectively. When combined with C3 toxin treatment, the reduction in survival was more pronounced (Figure 1A), with a fold decrease of 2, 26, and 13, respectively. The same was observed with the knockdown of RhoA and RhoB (Figure 1B), and in the subline RhoA-N19 (Figure 1A). Soft-agar assays confirmed that UVA, UVB and UVC irradiation decreased HeLa cells survival by 2. 9-, 4. 8-, and 12-fold, respectively, effects again markedly enhanced by the RhoA inhibition with C3 toxin (Figure 1C). The effect of RhoA *LoF* in survival was observed for the three UV wavelengths, even working with low doses of radiation, however, the observed reduction in survival was proportional to the higher radiation energy (and consequent to its shorter wavelength). Similarly, downregulation of RhoA activity also interferes with cell proliferation in response to UV-radiation (Figure 1D). Growth curves corroborate the survival data showing that the combined treatment of Rho *LoF* with UV exposure, especially UVC, almost completely abolished the proliferation of HeLa cells. UV-light treatments concomitantly affected cell migration of the HeLa and the RhoA-N19 subline (Supplementary Figures 2A,B). RhoA-V14 subline, that stably overexpresses the constitutively active RhoA-V14 mutant (Gly to Val substitution in position 14), and exhibits high levels of RhoA-GTP form, was used here as additional control. Both HeLa and RhoA-V14 cells showed similar motility capacity under UV stress, whilst RhoA-N19 cells had their migration markedly compromised. RhoA *LoF* impaired the migration of RhoA-N19 cells after UV-radiation even in the presence of Mitomycin C compound, used in order to eliminate possible cell proliferation interfering effects (Supplementary Figure 2C). UVC-radiation reduces stress fibers formation and stimulates actin protrusion formation in HeLa cells, but these morphological changes expectedly recovered 6 h after irradiation. However, the C3 toxin inhibition worsens this phenotype that persisted up to 6 h after UV-stress (Supplementary Figure 2D).

### HeLa Cells Present Lower Cell Cycle Arrest, Senescence and Apoptosis When Compared to RhoA LoF Condition and Also Combined With UV Exposure

**FIGURE 2:**
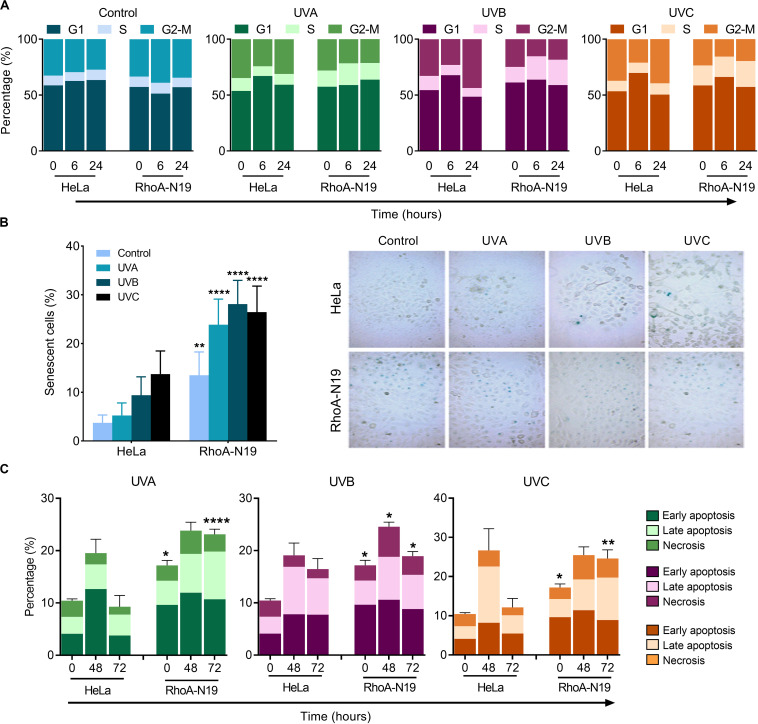
Cell cycle transitions and cell death mechanisms of asynchronous HeLa cells are affected by RhoA *LoF* and also subsequent UV-irradiation. **(A)** G1/S arrest is observed in the RhoA-N19 clones 6–24 h after UV radiation. **(B)** Cellular senescence associated to β-Galactosidase (SA-βGal) clearly distinguishes the cellular arrest induced by UV when HeLa cells are RhoA depleted. **(C)** Apoptosis analyses of HeLa cells by incubation with Annexin-V/PI showed only a discrete late and early apoptosis in cells with RhoA *LoF*. Graphs show mean ± SD of three independent experiments. Two-way ANOVA: (*) 0.01 ≤ *p* < 0.05, (**) 0.001 ≤ *p* < 0.01, and (****) *p* < 0.0001.

Analyses by flow cytometry were performed to investigate the roles of RhoA in cell cycle progression after UV-stress. Asynchronous population of HeLa cells showed G1-phase arrest 6 h after UV-radiation that was recovered 24 h following the stress. However, RhoA-N19 cells showed a strong and persistent S-phase arrest (and a discreet G1-phase arrest, especially after UVB and UVC) until 24 h after UV-radiation (Figure 2A and Table 2). This persistent cell cycle arrest can be associated with the impaired proliferation of RhoA-N19 cells (Figure 1D). A high% population of senescent cells was observed for the RhoA-N19 subline at the control condition (3 times higher than the parental cells) that was further increased after UV-radiation, reaching approximately 30% of senescent cells (Figure 2B). Autophagic cell death was checked by immunofluorescence and immunoblotting assays using the LC3B I/II autophagic marker, however, no signals of autophagy were observed in presence or absence of RhoA activity in HeLa cells after UV-radiation treatment (Supplementary Figure 3). Apoptosis verification by flow cytometry using Annexin-V and PI staining (Figure 2C and Table 3) revealed an increase in late and early apoptosis for HeLa cells 48 h after UV exposure. This increment was greater in early apoptosis after UVA, whereas after UVB and UVC, late apoptosis showed a greater increase. Cell death by necrosis did not change significantly over time after UV-stress. For HeLa cells, the levels of both early and late apoptosis almost returned to baseline 72 h after UV-stress. On the other hand, in RhoA-N19 cells, apoptosis levels were already higher even at basal condition, indicating a greater instability of this subline caused only by RhoA *LoF*. Apoptosis and necrosis were further increased by UV-radiation, with high levels of early and late apoptosis remaining up to 72 h, which again suggests RhoA as being directly relevant to cellular responses to UV-induced DNA lesions.

**TABLE 2:** Distribution of HeLa cells and RhoA-N19 clone on the cell cycle phases after UV-radiation.

	HeLa cells						
	
	Time after UV	G1 phase		S phase		G2/M phase	
				
		Mean (%)	*SD*	Mean (%)	*SD*	Mean (%)	*SD*
Control	0 h	58.8	± 7.2138	8.7	± 1.5576	32.5	± 5.6562
	6 h	62.6	± 4.1489	7.9	± 0.1889	29.5	± 3.9600
	24 h	63.5	± 3.1836	9.1	± 0.0139	27.3	± 3.1975
UVA	0 h	53.7	± 5.3398	11.6	± 0.1158	34.7	± 5.2241
	6 h	67.1	± 6.3992	8.6	± 2.0548	24.3	± 4.3443
	24 h	59.3	± 12.9787	9.6	± 3.6040	31.1	± 9.3747
UVB	0 h	54.5	± 1.9321	12.7	± 0.5059	32.9	± 2.4380
	6 h	67.8	± 1.4074	9.1	± 0.5800	23.1	± 0.8274
	24 h	48.5	± 7.3411	7.7	± 3.7048	43.7	± 11.0459
UVC	0 h	53.4	± 0.5980	9.3	± 1.3619	37.3	± 0.7639
	6 h	69.8	± 1.4634	9.2	± 0.3264	20.9	± 1.1370
	24 h	50.5	± 8.6231	10.2	± 6.5840	39.3	± 15.2071

	**RhoA-N19 subline**						

Control	0 h	56.5	± 16.0398	9.1	± 5.7417	32.9	± 23.8820
	6 h	50.4	± 16.7144	9.5	± 2.8638	38.3	± 22.0509
	24 h	56.3	± 18.7771	8.3	± 1.9021	34.0	± 22.6575
UVA	0 h	60.9	± 10.9644	15.4	± 4.0654	29.6	± 18.1865
	6 h	64.1	± 7.9707	21.0	± 7.7800	23.5	± 1.5481
	24 h	66.1	± 0.9394	15.4	± 4.3350	22.0	± 7.3714
UVB	0 h	63.2	± 7.6640	14.4	± 3.7733	25.6	± 11.5920
	6 h	68.4	± 5.7848	22.3	± 6.8518	16.4	± 2.0295
	24 h	65.7	± 3.1398	25.1	± 7.9442	20.4	± 8.8438
UVC	0 h	62.3	± 4.2537	19.0	± 5.6311	25.0	± 11.3371
	6 h	69.2	± 8.6929	18.9	± 6.0548	16.3	± 2.3976
	24 h	62.4	± 11.6529	25.3	± 10.6502	21.2	± 1.1081

**TABLE 3:** Apoptosis and necrosis levels exhibited by HeLa and HeLa-N19 cells after UV-induced DNA damage.

	HeLa cells								
	
	Time after UV	Early apoptosis		Late apoptosis		Necrosis		Total apoptosis	Total death
				
		Mean (%)	*SD*	Mean (%)	*SD*	Mean (%)	*SD*		
UVA	0 h	4.1	± 11.2964	3.2	± 10.9524	3.1	± 10.3564	7.3	10.4
	48 h	12.7	± 12.6518	4.7	± 0.7754	2.1	± 2.6676	17.4	19.5
	72 h	3.8	± 0.3099	4.0	± 0.6240	1.5	± 2.1483	7.8	9.3
UVB	0 h	4.1	± 1.2964	3.2	± 0.9524	3.1	± 0.3564	7.3	10.4
	48 h	7.8	± 2.4421	9.1	± 1.7619	2.2	± 2.3715	16.9	19.1
	72 h	7.7	± 2.0888	7.0	± 1.6089	1.8	± 2.0005	14.7	16.5
UVC	0 h	4.1	± 1.2964	3.2	± 0.9524	3.1	± 0.3564	7.3	10.4
	48 h	8.2	± 3.4118	14.4	± 1.8561	4.1	± 5.5112	22.6	26.7
	72 h	5.5	± 0.4636	4.7	± 1.4568	2.0	± 2.2504	10.2	12.2

	**RhoA-N19 subline**								

UVA	0 h	9.6	± 2.7183	4.6	± 0.5581	2.9	± 0.9326	14.3	17.2
	48 h	12.0	± 0.7973	7.4	± 0.7466	4.4	± 1.5948	19.4	23.8
	72 h	10.7	± 2.7389	9.1	± 1.8333	3.3	± 0.9780	19.8	23.1
UVB	0 h	9.6	± 2.7183	4.6	± 0.5581	2.9	± 0.9326	14.3	17.2
	48 h	10.6	± 0.8066	8.2	± 0.9969	5.7	± 0.8848	18.8	24.6
	72 h	8.8	± 2.0773	6.5	± 1.9255	3.6	± 0.8836	15.4	18.9
UVC	0 h	9.6	± 2.7183	4.6	± 0.5581	2.9	± 0.9326	14.3	17.2
	48 h	11.4	± 1.9519	7.9	± 1.2095	6.2	± 2.0747	19.3	25.5
	72 h	8.9	± 1.6823	10.8	± 1.9166	4.9	± 2.2018	19.7	24.6

### Different UV Treatments Indistinctly Provoke a Delayed Repair of Global DNA Strand Breaks in HeLa Cells Submitted to RhoA LoF

**FIGURE 3:**
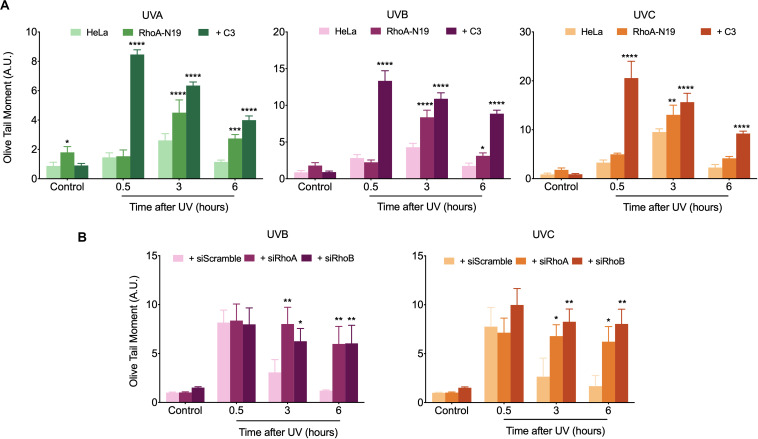
In HeLa cells under Rho inhibition and subsequent UV exposure, the DNA strand breaks strongly accumulate over time. An increasing UV-induced DNA fragmentation and impaired repair is observed in HeLa cells under Rho inhibition by the C3 toxin and overexpression of the RhoA-N19 mutant **(A)**, as well as in cells under RhoA and RhoB knockdown by specific siRNAs **(B)**, as measured by alkaline comet assays up to 6 h after UV radiation treatments. Graphs show mean ± SD of three independent experiments. Two-way ANOVA: (*) 0.01 ≤ *p* < 0.05, (**) 0.001 ≤ *p* < 0.01, (***) 0.0001 ≤ *p* < 0.001, and (****) *p* < 0.0001.

Comet assay was initially used to investigate the possible RhoA involvement in the global repair of DNA strand breaks, in different time-points after exposure to UV-radiation, which can cause direct or many indirect DNA fragmentation (Figure 3). HeLa cells submitted to RhoA *LoF* by different methods displayed similar profiles of DNA breaks after UV-light exposure: in the parental cells, the fragmented DNA levels were higher in the time-point of 30 min and returned to basal level 6 h after irradiation. On the other hand, Rho *LoF* by C3 toxin inhibition or the deficient Rho-N19 clone (less pronounced) increased dramatically the levels of fragmented DNA in HeLa cells right after all three UV wavelengths, what suggests an increase in radiosensitivity (Figure 3A). Moreover, HeLa cells under RhoA *LoF* also presented an accumulation of DNA fragmentation up to 6 h after UV treatments, being unable to recover to the lower basal levels of fragmentation without stress. The rate of DNA breaks repair was determined by a linear regression transformation (Supplementary Table 1). To simplify the analysis comprehension, it was assumed an approximation in which the speed of repair was constant – and thus directly proportional to the absolute value of the slope. Therefore, this regression shows that RhoA *LoF* by itself also decreases the repair rate. Similarly, the knockdown of RhoA and RhoB also increased the DNA damage and impaired the DNA breaks repair after UVB or UVC exposure (Figure 3B). Despite both siRNA presented very similar effects, RhoA knockdown seems to be more relevant in response to UVC radiation compared to RhoB knockdown, which was more evident in response to UVB radiation, as also evidenced by the reduction in the repair rates (Supplementary Table 1). Scramble siRNA behaved very similarly to parental Hela cells in spite of the interferences expected by this control.

### The CPD Levels in HeLa Cells Are Consistently Elevated Under RhoA LoF

**FIGURE 4:**
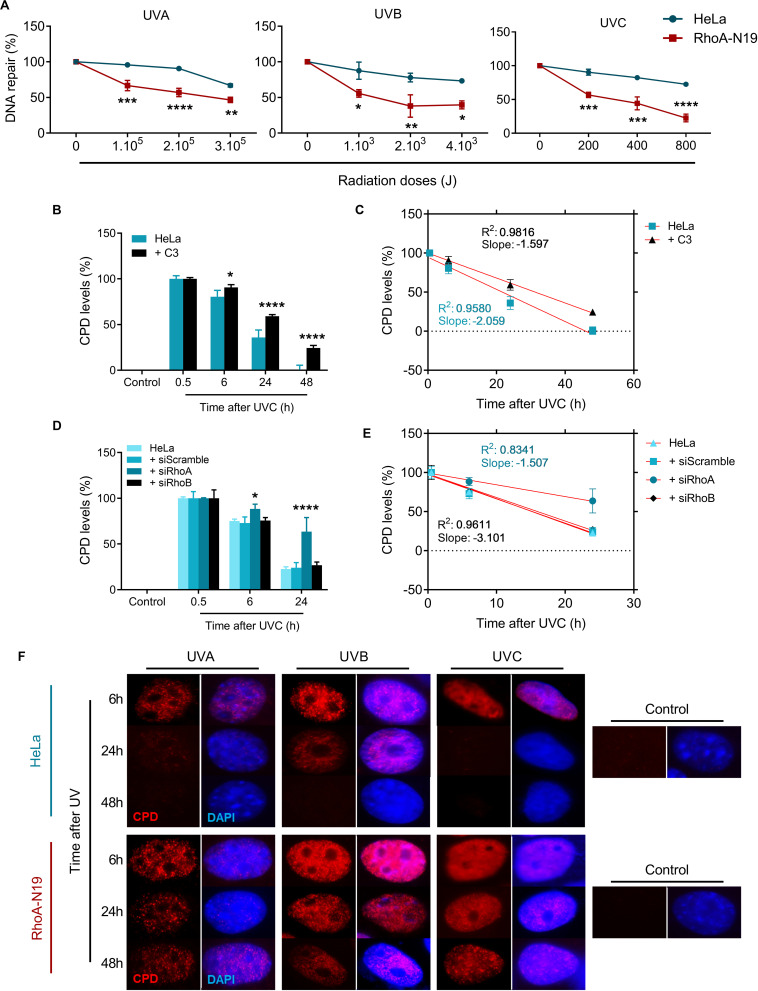
The repair of either exogenous or endogenous cyclobutane pyrimidine dimer (CPD) lesions is impaired in HeLa cells under RhoA *LoF*. **(A)** HeLa and RhoA-N19 cells were transiently transfected with exogenous plasmidial DNA previously irradiated with increasing doses of UVA, UVB, and UVC. The repair of UV-induced lesions was monitored by host cell reactivation (HCR) assay and RhoA-N19 subline presented the lowest repair. HeLa cells submitted to C3 toxin inhibition **(B)**, the RhoA/RhoB knockdown **(D)** or even overexpressing the RhoA-N19 mutant **(F)** were UV-irradiated and analyzed as to the levels of CPDs in the genomic DNA at different times after the damage by using the anti-CDP immuno-based techniques (slot-blot or immunofluorescence assays). Different RhoA *LoF* strategies led to similarly high and persistent levels of CPD even 48 h after UV-stress. Linear regression graphs showing the slope and R^2^ from slot-blot of cells submitted to C3 toxin inhibition **(C)** and RhoA/RhoB knockdown **(E)** demonstrate a proportionally decrease in the speed of CPD repair by the RhoA *LoF*. Graphs show mean ± SD of three independent experiments. Two-way ANOVA: (*) 0.01 ≤ *p* < 0.05, (**) 0.001 ≤ *p* < 0.01, (***) 0.0001 ≤ *p* < 0.001, and (****) *p* < 0.0001.

To investigate if RhoA activity modifies the endogenous capacity of repairing UV-induced lesions on DNA, Host-Cell Reactivation (HCR) assays were performed using a firefly luciferase gene reporter (Figure 4A). For this assay, UV-treated luciferase plasmids were transfected into HeLa cells and in RhoA-N19 clone. The bioluminescence detected by the reactivation of luciferase expression was directly correlated to the cells ability to repair UV-promoted lesions on exogenous DNA through endogenous enzymatic machinery. The RhoA-N19 subline presented a markedly reduced capacity to repair exogenous UV-damaged DNA compared to control cells, independently on the UV-light wavelength and, therefore, indistinctly of the lesion types (direct from UVC, or more indirect and oxidative from UVA/UVB) (Figure 4A). Additionally, immunoassays for the direct quantification of Cyclobutane Pyrimidine Dimers (CPDs), a specific and highly toxic DNA lesion promoted by all three UV-radiation wavelengths, were performed to investigate the effects of RhoA *LoF* in the repair of these sites (Figure 4 and Supplementary Figure 4). Slot-blot assays were performed using a specific antibody that detects CPDs in genomic DNA samples extracted from cells after UV exposition. The CPD levels peaked 0.5 h after UVC exposure in parental HeLa cells, which was able to almost completely repair them up to 24 h, while Rho inhibition by C3 toxin (Figure 4B and Supplementary Figure 4A) or the RhoA knockdown (Figure 4D and Supplementary Figure 4B) strongly sensitized the cells by increasing the CPD lesions and delaying their repair 48 h after the treatment. RhoB knockdown did not affect the efficacy or the speed of CPD repair, as well as the scramble siRNA, very likely because this Rho isoform is not so necessary for a NER-dependent repair of these lesions. The rate of CPD lesions repair was also correlated to the absolute value of the slope curve through linear regression transformations. Rho inhibition by C3 toxin decreases the CPD repair rate compared to HeLa cells (Figure 4C and Supplementary Table 2). Similarly, RhoA knockdown also strongly decreased the CPD repair rate (Figure 4E and Supplementary Table 2). Compatible results were observed for the RhoA-N19 subline submitted to UVA, UVB, or UVC radiation, that is, the CPD levels were kept high even at 48 h after stress, more or less correspondingly to the UV radiation potency (Figure 4F).

### The Phosphorylation of Classical DDR Proteins Is Affected by RhoA LoF

**FIGURE 5:**
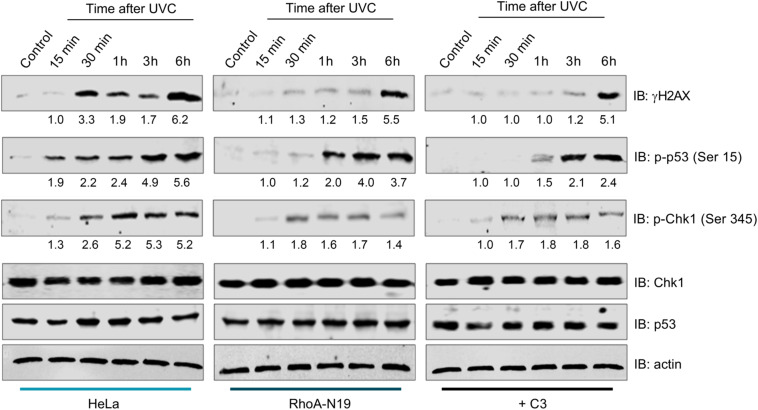
Inhibition of RhoA activity affects DDR signaling through the phosphorylation of sensor proteins after UVC radiation. The expression levels and phosphorylation kinetics for the proteins pH2AX-Ser139 (γH2AX), pChk1-Ser345 and pp53-Ser15 in RhoA proficient (parental HeLa) and deficient (HeLa RhoA-N19 mutant and HeLa + C3 toxin) cells after irradiation with 6 J/m^2^ UVC were assessed by immunoblotting. The blots quantification is numerically shown under each band. The shown blots are representative of three independent experiments.

To investigate whether RhoA *LoF* only affects a specific repair pathway (NER) or a more general pathway triggered for sensing general DNA damage (DNA damage response pathway, DDR), we performed immunoblottings to check the phosphorylation status of proteins involved in DDR (Figure 5). The kinetics of the histone variant H2AX-Ser139 phosphorylation, commonly assumed as a DNA strand breaks sensor, started 15 min delayed in parental HeLa cells and peaked 6 h after UVC. Under RhoA *LoF* a strong signal of H2AX phosphorylation was only observed 6 h after UVC treatment. UVC radiation promoted Chk1-Ser345 phosphorylation in HeLa cells starting 15 min and reaching a plateau up to 6 h after irradiation. RhoA *LoF*, either by the C3 toxin treatment or the Rho-N19 mutant cells, showed similar Chk1 phosphorylation kinetics, but strikingly attenuated after the treatment (Figure 5). Interestingly, the high and growing levels of p53-Ser15 phosphorylation in the control HeLa cells in response to UVC-induced DNA damage were progressively attenuated by both forms of RhoA *LoF* (Figure 5).

### Survival and Proliferation of NER- and TLS-Deficient Cells Are Still Affected by Rho LoF

**FIGURE 6:**
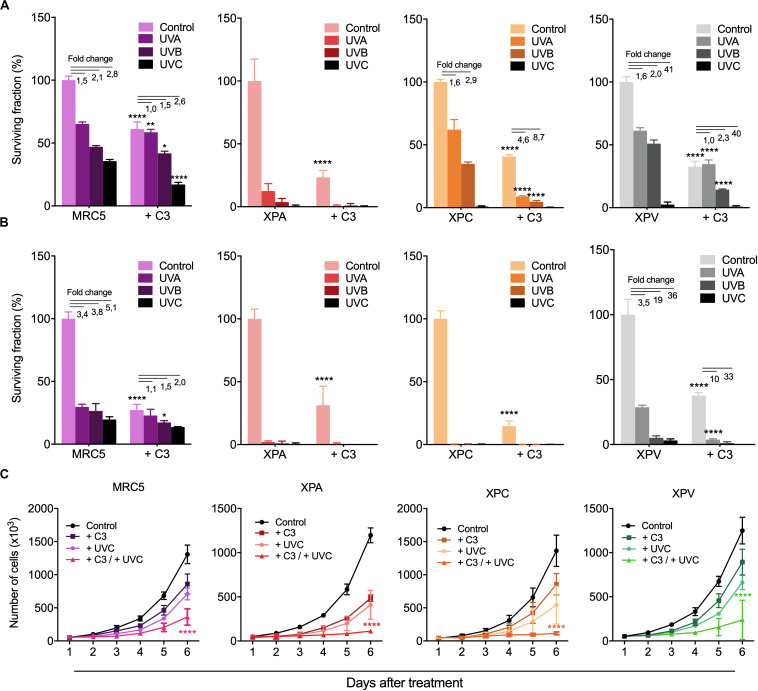
RhoA *LoF* strongly decreased survival and proliferation of NER- deficient cells compared to TLS-deficient and fibroblast cells in response to UV-radiation. Mid- and long-term proliferation assays for MRC5 fibroblasts, NER-deficient cells (XPA and XPC) and TLS-deficient cells (XPV) previously treated with C3 toxin and subsequently exposed to UV-radiation measured by 2D clonogenic assay **(A)**, 3D soft-agar colony formation assay **(B)** and cell growth curves **(C)**. Graphs represent mean ± SD from three independent experiments. Two-way ANOVA: (*) 0.01 ≤ *p* < 0.05, (**) 0.001 ≤ *p* < 0.01, and (****) *p* < 0.0001.

The effects of RhoA *LoF* in both DDR and repair of different UV-promoted lesions suggest possible implications of RhoA in the regulation of NER pathway. Therefore, skin-derived cells from Xeroderma Pigmentosum patients were used to investigate the interplay between RhoA and NER proteins. Two different NER-deficient cell lines (named XPA and XPC, which lack the XPA and the XPC proteins, respectively) and one DNA translesion synthesis (TLS)-deficient cell line, also known as XP variant or XPV (due to the deficiency of the Polη gene) were compared to a normal lung fibroblast cells (MRC5, used as control because its proficiency in NER and TLS pathways). All cells were submitted to RhoA *LoF* by using the C3 toxin (Supplementary Figure 5) and subsequently to UV treatments. UVA, UVB, and UVC radiation decreased clonogenic survival of NER-proficient cell lines MRC5 and XPV, with only a discrete additive effect in the absence of RhoA activity (treated with C3 toxin) (Figure 6A). However, all three UV-radiation wavelengths drastically decreased survival of the NER-deficient cells in 2D colony formation assays. RhoA inhibition enhanced XPA and XPC proteins-deficiency leading to a more drastic cell survival rates (Figure 6A). As proof-of-concept, these experiments were repeated through 3D colony formation assays in soft-agar matrix (a structured matrix mimicking *in vivo* tissue microenvironments) and showed that UV-radiation further compromised survival when combined with RhoA *LoF*, again with marked increase in XPA and XPC-deficient cells, but less evident in MRC5 and XPV cells (Figure 6B). The anti-survival association between NER and RhoA deficiency were corroborated by cell proliferation curves of NER- and TLS-deficient cells in response to UV-radiation (Figure 6C). It was observed that either UVC radiation or RhoA inhibition isolated treatments decreased proliferation of all cells, but the combined treatments led to a potentialized anti-proliferative effect, again clearly more evident in NER-deficient cells (Figure 6C).

### Rho LoF Sensitizes Even More XP Cells to UV-Radiation by Pushing DNA Damage to High Levels and Also Exacerbating DDR Pathway Responses

**FIGURE 7:**
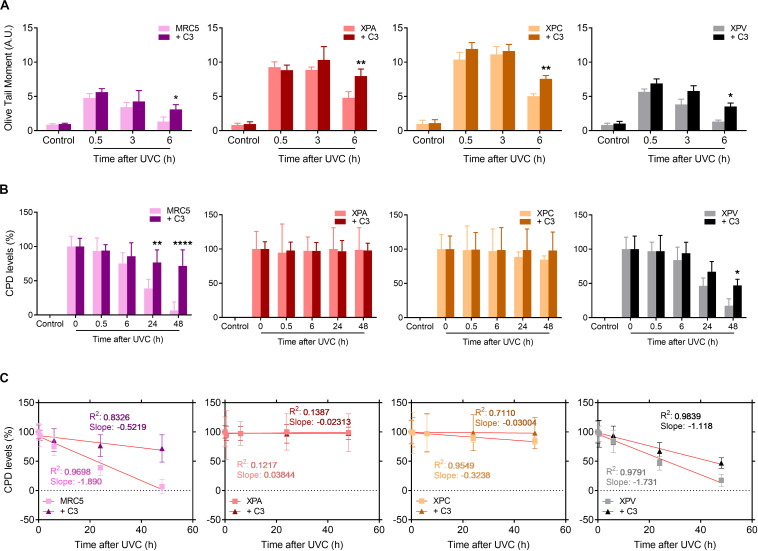
UV-radiation elevates the levels of DNA strand breaks and CPD lesions in NER- and TLS-deficient cells, effects that are worsened by the Rho *LoF*. **(A)** Alkaline comet assays showed the DNA fragmentation (measured by OTM parameter) in MRC5 fibroblasts, NER- and TLS-deficient cells submitted to UVC radiation is increase by previous RhoA inhibition with C3 toxin. **(B)** The levels of cyclobutane pyrimidine dimer (CPDs) lesions in the genomic DNA of NER- and TLS-deficient cells exposed to UVC is persistently high under previous RhoA *LoF* until 48 h after irradiation, as measured through immuno slot-blot assays (Supplementary Figure 6). **(C)** Linear regression transformations of graphs displayed in **(B)** show a decrease in the speed of CPD repair in both MRC5 and XPV cells. Graphs represent mean ± SD from six independent experiments. Two-way ANOVA: (*) 0.01 ≤ *p* < 0.05, (**) 0.001 ≤ *p* < 0.01, and (****) *p* < 0.0001.

Since RhoA activity is necessary for cell proliferation and survival after the deleterious effects of UV-radiation, especially for those with serious DNA repair defects, we moved to investigate these cells ability to repair UV-induced DNA lesions specifically through the NER pathway by performing alkaline comet and immuno-slot-blot assays in different time-points after UV exposure (Figure 7). All four cell lines displayed a maximum of DNA strand breaks 30 min after UV exposure, but NER-deficient cells expectedly showed to be more sensitive to DNA breaks accumulation. MRC5 fibroblasts and XPV-deficient cells present an OTM index close to 5 whereas XPA and XPC cells the OTM is close to 10. Besides that, while MRC5 fibroblasts and TLS-deficient cells display reduced DNA strand breaks already by 3 h after UV and an almost complete repair at the 6 h time-point, the NER-deficient cells only show signs of decrease in DNA breaks 6 h after UV (Figure 7A). The speed of strand breaks repair, also analyzed by the linear regression transformations, shows that C3 toxin significantly decrease the slope, and consequently, the repair rate in all four cell lines (Supplementary Table 3). Therefore, the inhibition of Rho had two distinct effects in these cells: in MRC5 fibroblasts (and less in XPV-deficient cells), Rho *LoF* increased the amount of DNA breaks and delayed the repair similarly to what was observed for HeLa cells (Figure 3); in NER-deficient and Rho-proficient cells, Rho *LoF* did not increase the levels of DNA breaks (previously with high damage), but significantly delayed the repair as observed by the greater amount of DNA breaks 6 h after UVC (OTM ∼ 50% higher).

Focusing on NER-dependent repair of specific DNA damage promoted by UV, slot-blots for CPD detection showed high levels of this lesion right following UVC exposure (0 h) in all cells. Therefore, CPD lesions were almost completely repaired in MRC5 fibroblasts and XPV-deficient cells 48 h after UV-stress (Figure 7B and Supplementary Figure 6). The RhoA *LoF* by C3 toxin was able to accurately impair the CPDs repair along all time-points of kinetics, in both MRC5 fibroblasts and XPV-deficient cells. In NER-deficient cells, which are known to be unable to repair CPD damage, the levels of CPDs did not change throughout the entire experiment. Linear regression transformations also showed a decrease in CPD repair rate in MRC5 and XPV-deficient cells (Figure 7C), similarly to HeLa cells (Supplementary Table 2), but not in NER-deficient cells and especially for the XPA line (Supplementary Table 4). More interestingly, the RhoA *LoF* by C3 toxin *per se* increased the levels of CPD in all cells immediately after UVC-radiation, but especially and unexpectedly mostly in XPA, XPC and HeLa cells (Supplementary Figure 7). These data show that RhoA inhibition hypersensitizes cells to UV radiation, compromises the NER pathway functions and maintains high the CPD levels, further corroborating the lack of XPA or XPC proteins.

**FIGURE 8:**
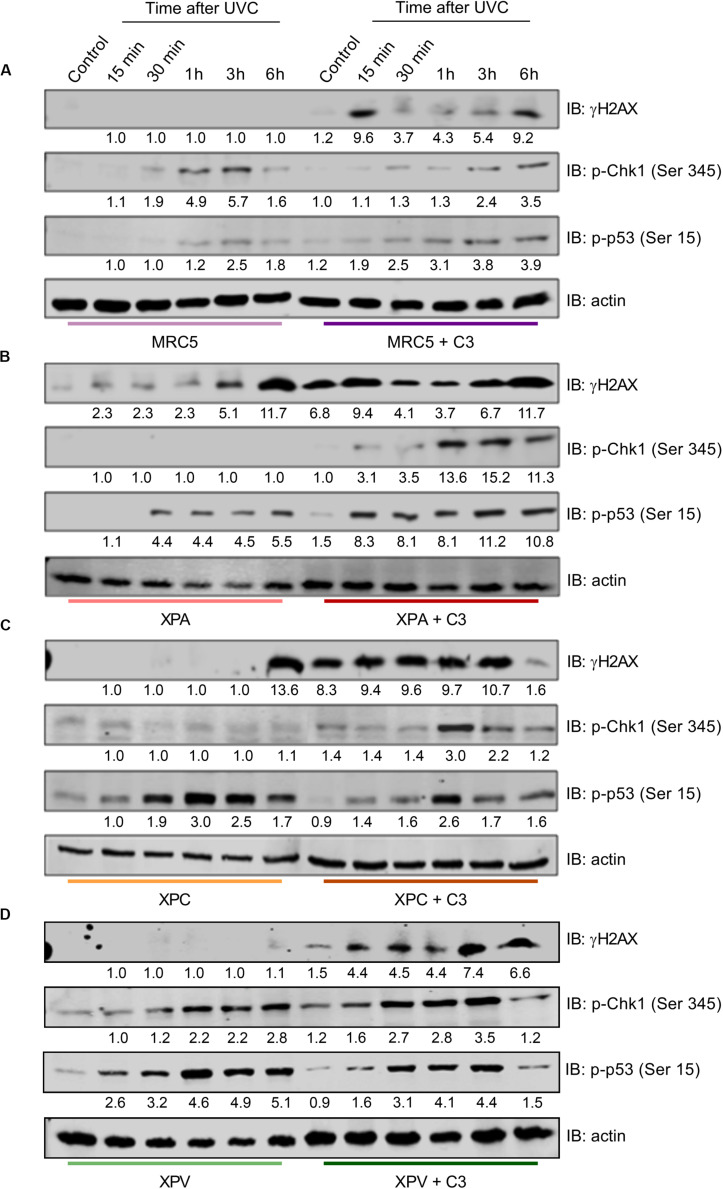
RhoA *LoF* differentially affects the phosphorylation of H2AX, Chk1, and p53 throughout the DDR signaling in NER- and TLS-deficient cells after UV-radiation stress. Immunoblottings showing the levels of expression and phosphorylation kinetics for Chk1, H2AX, and p53 in MRC5 **(A)**, XPA-deficient **(B)**, XPC-deficient **(C)**, and XPV-deficient **(D)** cells up to 6 h after the irradiation with 6 J/m^2^ UVC, without or with previous RhoA inhibition by C3 toxin. The blots quantification is numerically shown under each band. Blots are representative of three independent experiments.

Next, we verified the DDR signaling in response to UV radiation in NER- and TLS-deficient cells under RhoA *LoF* (Figure 8), since this would directly impact in the NER functioning. MRC5 normal fibroblasts did not show H2AX phosphorylation after UVC radiation. On the other hand, RhoA *LoF* promoted two peaks of H2AX-Ser129 phosphorylation, the first in 15 min and the other 6 h after UVC. Low phosphorylation of Chk1-Ser345 was detected in these cells in response to UVC, even with C3 toxin treatment, but with a delayed profile that persisted up to 6 h after treatment. The p53-Ser15 phosphorylation was slightly increased in MRC5 cells under RhoA *LoF* compared to normal RhoA activity, with a very similar profile observed for pChk1 (Figure 8A). NER-deficient cells showed comparable profiles of DDR proteins phosphorylation (Figures 8B,C). H2AX phosphorylation was triggered only 6 h after UVC radiation in both XPA and XPC cells. C3-driven RhoA *LoF* promoted an exacerbated H2AX phosphorylation, even in the control condition, which persisted up to 6 h for both cells (especially XPA compared to XPC). Phosphorylation of Chk1 in response to UVC irradiation was only detected in these two cells previously submitted to C3 toxin treatment and started late at approximately 1 h after the irradiation. The p53 phosphorylation profile was found practically the opposite in the NER-deficient cells: it was higher under RhoA *LoF* in XPA cells (with an attenuated profile under RhoA presence) and lower in XPC cells C3-treated (with an exacerbated profile under RhoA presence, but in a similar kinetics). TLS-deficient cells presented a similar behavior to MRC5 fibroblasts (Figure 8D). XPV cells only showed H2AX phosphorylation after UVC radiation when previously submitted to C3 toxin and reached a maximum in the late time-points. Phosphorylation of Chk1 was increased 30 min after UVC in control cells, persisting until 6 h after stress, while under RhoA *LoF* it was anticipated in the 15 min time-point after UVC, which only persisted until 3 h. Phosphorylation of p53 very similarly followed the kinetics of pChk1, in presence and absence of RhoA *LoF*, thus starting 15 min after UVC irradiation and with different duration, being this p53 phosphorylation ended earlier in XPV cells under Rho *LoF* (3 h after UVC).

## Discussion

In this work we identify Rho GTPases as unknown and underestimated regulators of NER pathway. We bring to attention that RhoA, RhoB and RhoC (RhoA/B/C) loss of function (*LoF*) impairs the survival and proliferation of HeLa cells after UV-stress very likely because of an inefficient ability to specifically repair direct and indirect UV-promoted DNA damage. We also demonstrated RhoA *LoF* affects the DDR signaling in a NER-dependent manner. Our data show that Rho *LoF* strongly sensitized HeLa cells to UV-radiation decreasing survival proportionally to the higher efficiency of Rho inhibition. For example, C3 toxin, that inhibits RhoA, RhoB and RhoC, displayed a more drastic impairment of survival than RhoA/B knockdown or RhoA-N19 overexpressing cells (Figure 1). Despite the high homology among them, the three Rho GTPases present distinct biological roles and share some similar functions in the regulation of actin cytoskeleton, besides to interact with some of the same effectors, but with different affinities (Wheeler and Ridley, 2004). Our findings show the *LoF* for all Rho was adverse to cell survival, indicating a mechanism that likely occurs through a common pathway regulated by the three Rho isoforms. Despite the different wavelengths (UVA, UVB, and UVC) UV-light decreased cell survival and proliferation of HeLa cells with a higher effect when combined with Rho *LoF*, however, these deleterious effects were proportional to higher radiation energy (Figure 1). UV-radiation generates distinct DNA lesions according to wavelengths, being UVA the most responsible for bases oxidation and single strand breaks (minor generation of CDP lesions), while UVB and UVC are the main cause of photoproducts formation (and less oxidative lesions by UVB) (Schuch et al., 2017). Due to ROS formation, UVA present the highest mutational capacity, but these mutations preferentially occur in non-transcribed strands, so these lesions can be tolerated by cells, with a lower effect in the survival. DNA photoproducts (mainly CPDs) were reported to be the major pre-mutagenic and genotoxic lesions, leading to higher mutagenic behaviors, cell cycle arrest and cell death (Rünger and Kappes, 2008; Schuch and Menck, 2010; Schuch et al., 2017; Mullenders, 2018). Therefore, the effects on survival observed here for the most energetic UV-light probably occurs due to the higher formation of these photoproducts.

Survival and proliferation data were corroborated by the cell cycle arrest in G1/S (with a small arrest in G1-phase only under UVB and UVC treatments) observed with RhoA *LoF* and subsequent UV exposure (Figure 2A). RhoA *LoF* also increased cell death after UV-stress through senescence and apoptosis mechanisms; senescent cells were more observed under UVB/UVC exposure, while apoptotic cells were found in all UV-light wavelengths. Necrosis was identified in higher levels especially under UVB and UVC stress (Figures 2B,C), but not autophagic death (Supplementary Figure 3) was detected. UV-radiation can induce G1/S arrest and apoptosis, as well senescence, by the modulation of p21^Waf1/Cip1^, p16 and p53 proteins, by driving cells to loss of replicative potential, increased SA-βGal activity and overexpression of senescence-associated genes (Chen et al., 2015; Toutfaire et al., 2017). RhoA pathway was also related to cell cycle progression and cell death: its inactivation regulates G1-arrest by increasing the cell cycle inhibitors p21^Waf1/Cip1^ and p27^Kip1^, whereas its activation by GAPs downregulation (ArhGAP11A and RacGAP1) leads to p27^Kip1^ and p21^Waf1/Cip1^-dependent cell cycle arrest, reduced phospho-Rb levels and increased senescence (Zhang et al., 2009; Haga and Ridley, 2016; Lawson et al., 2016). Other connections between genotoxic stress and Rho pathway on the regulation of cell cycle and death mechanisms have been demonstrated. For example, DNA damage was shown to induce actin reorganization influencing cell cycle arrest and subsequent apoptosis, and disruption of actin filaments (by Rho or actin inhibitors) also increases p21^Waf1/Cip1^ protein stability (Chang et al., 2015). RhoA activation, in response to DNA damage, leads to stress fiber formation and enhanced cell survival through p38 MAPK activation (Guerra et al., 2008). Thus, our data associated with previous ones from literature suggest that RhoA *LoF* synergizes the UV-promoted cell cycle malfunction, very likely through p21^Waf1/Cip1^-induced mechanisms of senescence and apoptosis.

RhoA/B/C have also been increasingly related to DNA repair mechanisms, since they were: (i) found activated in response to ionizing radiation (Dubash et al., 2011), (ii) transcriptionally induced by the formation of DSBs to affect Chk2 and H2AX phosphorylation status (Mamouni et al., 2014), and (iii) found activated in response to oxidative lesions such as 8-oxoG and its subsequent repair (Luo et al., 2014; Seifermann and Epe, 2017). Here we showed that Rho *LoF* strongly impaired the repair of UV-damaged DNA, especially the repair of CPDs and strand breaks. The latter occur due to either direct UV effects (minority) or indirect damage promoted secondarily through drastic distortions on DNA-helix and/or impaired DNA replication/transcription caused by CPDs, 6-4-PPs and oxidative lesions (Mullenders, 2018). From the comet assays we showed that Rho *LoF per se* is enough to impair the fragmented DNA repair, which can be additionally influenced by the different inhibition methods and/or UV wavelengths (Figure 3). Similar responses were observed for the repair of specific UV-induced lesions. For example, the host cell reactivation assays showed that RhoA *LoF* impacts on the endogenous capacity of repairing UV-specific lesions generated in an exogenous DNA plasmid (Figure 4A), and also significantly reduced the repair of CPD lesions, which persisted days after the stress (Figures 4B–F). Interestingly, the isoforms RhoA and RhoB play different roles on the DNA repair and also according to the different UV wavelengths: RhoA seems to be more necessary for the repair of UVC-induced DNA breaks and CPDs, while RhoB is apparently more relevant for the repair of UVB-induced DNA breaks, not significantly affecting the repair of CPDs. UVB and UVC promote very similar effects on DNA, however, due to its shorter wavelength, UVC is more absorbed by the DNA and generates higher levels of CPD at low doses, consequently causing more DNA breaks (Foresti and Avallone, 2008). Some important correlations between Rho GTPases and DNA strand breaks repair have emerged in the last few years and possibly can help us to explain these results. For example, the expression and activity of RhoB, but not RhoA, was rapidly induced in response to CPT-induced DSBs, while its knockdown impairs the repair of these lesions in mouse embryonic fibroblasts (Mamouni et al., 2014). By contrast, RhoA has also been linked to DNA repair machinery once its higher activity is directly correlated to higher DNA repair capacity of the cells (Sahai et al., 2001; Luo et al., 2014).

Rho *LoF* seems to increase the sensitivity of HeLa cells to UV-radiation due to increasing levels of DNA damage (strand breaks and CPD lesions) (Figures 3, 4) caused by the deregulation of F-actin dynamics. Perturbations of these filaments, spread all over the cell, can in fact overexpose intracellular components allowing these biomolecules to absorb more radiation and, consequently, to present higher basal damage. Is known for several years that Cytochalasin B, a drug that prevents the actin polymerization, increases radiosensitivity to ionizing radiation in a ECM-dependent manner (Stevenson and Lange, 1997). Disruption of the actin network also impairs the transport of actin-dependent proteins and organelles, affecting the transport of proteins/complexes involved in DNA damage response and repair, as well as those involved in chromatin remodeling. It was demonstrated recently that actin dynamics is crucial to RPA recruitment to DSB sites after Doxorubicin treatments (Pfitzer et al., 2019). Additionally, the overexpression of Cofilin-1, a downstream component of RhoA pathway, impairs both actin polymerization and DSB repair leading to increased radiosensitivity (Chang et al., 2015). Nuclear F-actin and myosin (as in stress fibers) were identified to participate of the heterochromatin remodeling in response to DSBs, regulating the repair of these lesions through the HR pathway (Caridi et al., 2018). Although without evidences of molecular mechanisms, both RhoA and RhoB seem to be necessary for the repair of general UV-induced damage, each isoform contributing distinctly to specific DNA lesions; RhoA being possibly more involved in NER pathway and RhoB more relevant for pathways of strand breaks repair. Furthermore, the participation of one or the other Rho GTPase may also be attributed to any of its specific effectors, which contribution to genomic stability mechanisms is still totally unknown.

Other results reinforced the correlation between typical Rho GTPases and NER pathway overall contributing to the maintenance of genomic stability. For example, is the confirmation that Rho *LoF* impairs the DDR signaling in HeLa cells by decreasing phosphorylation levels of H2AX-S139, Chk1-S345, and p53-S15 after UV-exposure (Figure 5). It was previously reported that Rac1 inhibition reduces H2AX, Chk1, and p53 phosphorylation levels in a ATM/ATR-dependent pathway following ionizing radiation and Doxorubicin treatments (Fritz and Henninger, 2015), similarly to what we observed here in UV-treated HeLa cells under different Rho *LoF* conditions, which also suggests RhoA as a regulator of DNA repair in HeLa cells by modulating DDR mechanisms (Figure 5). Our results indicate that the RhoA *LoF* is possibly promoting two mechanisms: (i) facilitating and increasing UV-induced DNA breaks, and (ii) preventing an adequate signaling of damage recognition and, consequently, the correct activation of repair machineries. If these assumptions are correct, cells under RhoA *LoF* carry DNA strand breaks but the DDR pathway cannot be properly activated to signal these damage installation.

Nevertheless, there are no molecular mechanisms correlating Rho and NER pathway and, despite DDR pathway seems to be a potential mediator between them, we attempted to indirectly explore some mechanisms or molecular targets. NER dysfunction causes a syndrome called Xeroderma Pigmentosum, where the lack of any one of the eight NER and TLS genes (XPA-XPG and XPV) compromises DNA damage repair and tolerance at different stages, leading to a higher sensitivity to UV-light and increased susceptibility to skin cancer (Oh et al., 2011). Our results showed that Rho *LoF* enhances UV-induced stress in XPA- and XPC-deficient cells, differently from XPV-deficient and normal MRC5 fibroblasts, and drastically reduce their cell survival and proliferation (Figure 6). Interestingly, XPA protein presents a distinct cell cycle-dependent localization, being retained at the cytosol in G1-phase, while it is mostly nuclear in G2-phase, independently of UV-damage. Under UV-stress, this protein translocates to the nucleus in S-phase through a ATR and p53 dependent mechanism, which is facilitated by importin-α4 in a process dependent on a unknown GTPase (Li et al., 2013; Musich et al., 2017). In other report, TGFβ leads to cell cycle arrest and inhibits proliferation through RhoA/ROCK pathway and induces nuclear localization of ERCC1/XPA and ERCC1/XPF complex (Bhowmick et al., 2003; Zheng et al., 2019). XPA roles in cell cycle progression were observed by the downregulation of the XPA-binding protein 2 (XAB2) affecting the transcription of mitotic related genes, including the centrosome-associated gene E (CENP-E) (Hou et al., 2016). On the other hand, Rho pathway is related to centrosome organization through the effector mDia2 by maintaining the correct levels of CENP-A at the centrosomes (Liu and Mao, 2017), therefore highlighting a potential correlation between Rho pathway and XPA protein, even independently of NER pathway, thus contributing for the understanding of cellular responses reported here. Indeed, Rho *LoF* is still able to increase sensitivity of NER-deficient cells to UV radiation effects by elevating the levels of DNA fragmentation and accumulation of CPD lesions (Figures 6,7 and Supplementary Figures 6, 7). However, the incapacity of XP-deficient cells to repair CPD lesions and recover the basal levels remains unaffected by the RhoA *LoF* (Figure 7), therefore sustaining the hypothesis of this work that RhoA GTPase mediates NER pathway function.

Intriguingly the DDR signaling presented a distinct regulation between NER-deficient cells, TLS-deficient cells, and normal MRC5 fibroblasts, all differing from of what we observed in HeLa cells (Figure 8 vs. Figure 5). XPA- and XPC-deficient cells exhibited a quite delayed γH2AX phosphorylation in response to UV-light, while Rho *LoF* strongly increases this signal. MRC5 and TLS-deficient cells do not show any significant phosphorylation of H2AX after UV-exposure, which was only raised by RhoA *LoF*. Phosphorylation of Chk1-S354 after UV exposure was also only detected in NER-deficient cells under RhoA *LoF*, with a slight increase in fibroblast and XPV-deficient cells. The p53-S15 phosphorylation proved to be opposite between NER-deficient cells under RhoA *LoF*: it was much higher in XPA cells than in XPC cells, whereas it was also higher in XPV compared to MRC5 cells. Interestingly this very unusual phospho-p53 regulation proved to be a sensitive, complex and non-understood mechanism between the Rho-DDR-NER pathways. And, as discussed before, Rho GTPases implications in DDR regulation would certainly affect regulation of NER proteins and NER complexes assembly. Another good example of this complexity is that XPC deficiency upon Cisplatin treatments was shown to reduce BRCA1 levels leading to a persistent activation of ATM-Chk1/Chk2 and prolonged G2/M arrest, being the elevated γH2AX levels an indicative of higher number of non-repaired DSBs (Wang et al., 2019). ATR and ATM activation and accumulation under UVR-induced damage depends on DDB2, XPC and XPA proteins, suggesting that the assembly of an active NER complex is essential for ATR and ATM recruitment (Ray et al., 2016). These two proteins have also specific roles in cell protection and repair/tolerance of ROS-induced DNA damage. NER-deficient cells were hypersensitive to photoactivated methylene blue and also presented more γH2AX-stained nuclei and G2/M arrest (Maria Berra et al., 2013). Inhibition of RhoA/ROCK pathway increase intracellular ROS levels in melanoma cells through Rac1 activation, and also increase pATM, p-p53 and γH2AX levels without other external genotoxic stress source. The RhoA/ROCK inhibition also triggers the transcription of p53-activated genes involved in ROS metabolism and DNA response (Herraiz et al., 2016), that could explain the higher activation of γH2AX even without UV-exposure in NER-deficient cells under Rho *LoF* (that increase intracellular ROS levels). In sum, our findings here bring to the light a new and surprising interplay between Rho GTPases, DDR and NER pathways, helping to elucidate a more robust mechanism of genomic stability and launching new strategies to target these signaling pathways in translational medicine.

## Data Availability Statement

All datasets presented in this study are included in the article/Supplementary Material.

## Author Contributions

YM, GS, JO, and CR performed the experiments of this study. FF supervised and designed the study. YM, GS, JO, CR, and FF analyzed and interpreted the data. YM and FF wrote the manuscript. All authors contributed to the article and approved the submitted version.

## Conflict of Interest

The authors declare that the research was conducted in the absence of any commercial or financial relationships that could be construed as a potential conflict of interest.

## References

[B1] Al-Koussa H. El Atat O. Jaafar L. Tashjian H. El-Sibai M. (2020). The role of Rho GTPases in motility and invasion of glioblastoma cells. *Anal. Cell. Pathol. (Amst).* 2020:9274016. 10.1155/2020/9274016 32089990 PMC7013281

[B2] Bhowmick N. A. Ghiassi M. Aakre M. Brown K. Singh V. Moses H. L. (2003). TGF-β-induced RhoA and p160ROCK activation is involved in the inhibition of Cdc25A with resultant cell-cycle arrest. *Proc. Natl. Acad. Sci. U.S.A.* 100 15548–15553. 10.1073/pnas.2536483100 14657354 PMC307605

[B3] Borowicz S. Van Scoyk M. Avasarala S. Karuppusamy Rathinam M. K. Tauler J. Bikkavilli R. K. (2014). The soft agar colony formation assay. *J. Vis. Exp.* e51998. 10.3791/51998 25408172 PMC4353381

[B4] Caridi C. P. D’agostino C. Ryu T. Zapotoczny G. Delabaere L. Li X. (2018). Nuclear F-actin and myosins drive relocalization of heterochromatic breaks. *Nature* 559 54–60. 10.1038/s41586-018-0242-8 29925946 PMC6051730

[B5] Chang C.-Y. Y. Leu J.-D. Lee Y.-J. J. (2015). The actin depolymerizing factor (ADF)/Cofilin signaling pathway and DNA damage responses in cancer. *Int. J. Mol. Sci.* 16 4095–4120. 10.3390/ijms16024095 25689427 PMC4346946

[B6] Chen A. Huang X. Xue Z. Cao D. Huang K. Chen J. (2015). The role of p21 in *Apoptosis*, proliferation, cell cycle arrest, and antioxidant activity in UVB-irradiated human HaCaT keratinocytes. *Med. Sci. Monit. Basic Res.* 21 86–95. 10.12659/MSMBR.893608 25925725 PMC4427023

[B7] de Lima-Bessa K. M. Armelini M. G. Chiganças V. Jacysyn J. F. Amarante-Mendes G. P. Sarasin A. (2008). CPDs and 6-4PPs play different roles in UV-induced cell death in normal and NER-deficient human cells. *DNA Repair (Amst).* 7 303–312. 10.1016/j.dnarep.2007.11.003 18096446

[B8] Dubash A. D. Guilluy C. Srougi M. C. Boulter E. Burridge K. García-Mata R. (2011). The small GTPase RhoA localizes to the nucleus and is activated by Net1 and DNA damage signals. *PLoS One* 6:e17380. 10.1371/journal.pone.0017380 21390328 PMC3044755

[B9] Emri G. Paragh G. Tósaki Á Janka E. Kollár S. Hegedûs C. (2018). Ultraviolet radiation-mediated development of cutaneous melanoma: an update. *J. Photochem. Photobiol. B Biol.* 185 169–175. 10.1016/j.jphotobiol.2018.06.005 29936410

[B10] Espinha G. Osaki J. H. Costa E. T. Forti F. L. (2016). Inhibition of the RhoA GTPase activity increases sensitivity of melanoma cells to UV radiation effects. *Oxid. Med. Cell Longev.* 2016:2696952. 10.1155/2016/2696952 26823948 PMC4707346

[B11] Espinha G. Osaki J. H. Magalhaes Y. T. Forti F. L. (2015). Rac1 GTPase-deficient HeLa cells present reduced DNA repair, proliferation, and survival under UV or gamma irradiation. *Mol. Cell Biochem.* 404 281–297. 10.1007/s11010-015-2388-0 25758356

[B12] Foresti M. Avallone B. (2008). Only complete rejoining of DNA strand breaks after UVC allows K562 cell proliferation and DMSO induction of erythropoiesis. *J. Photochem. Photobiol. B Biol.* 90 8–16. 10.1016/j.jphotobiol.2007.05.008 18032060

[B13] Fritz G. Henninger C. (2015). Rho GTPases: novel players in the regulation of the DNA damage response? *Biomolecules* 5 2417–2434. 10.3390/biom5042417 26437439 PMC4693241

[B14] Guerra L. Carr H. S. Richter-Dahlfors A. Masucci M. G. Thelestam M. Frost J. A. (2008). A bacterial cytotoxin identifies the RhoA exchange factor Net1 as a key effector in the response to DNA damage. *PLoS One* 3:e2254. 10.1371/journal.pone.0002254 18509476 PMC2386254

[B15] Haga R. B. Ridley A. J. (2016). Rho GTPases: regulation and roles in cancer cell biology. *Small GTPases* 7 207–221. 10.1080/21541248.2016.1232583 27628050 PMC5129894

[B16] Han S. Arvai A. S. Clancy S. B. Tainer J. A. (2001). Crystal structure and novel recognition motif of Rho ADP-ribosylating C3 exoenzyme from *Clostridium botulinum*: structural insights for recognition specificity and catalysis. *J. Mol. Biol.* 305 95–107. 10.1006/jmbi.2000.4292 11114250

[B17] Herraiz C. Calvo F. Pandya P. Cantelli G. Rodriguez-Hernandez I. Orgaz J. L. (2016). Reactivation of p53 by a cytoskeletal sensor to control the balance between DNA damage and tumor dissemination. *J. Natl. Cancer Inst.* 108:djv289. 10.1093/jnci/djv289 26464464 PMC4712681

[B18] Hou S. Li N. Zhang Q. Li H. Wei X. Hao T. (2016). XAB2 functions in mitotic cell cycle progression via transcriptional regulation of CENPE. *Cell Death Dis.* 7:e2409. 10.1038/cddis.2016.313 27735937 PMC5133980

[B19] Ikehata H. Ono T. (2011). The mechanisms of UV mutagenesis. *J. Radiat. Res.* 52 115–125. 10.1269/jrr.10175 21436607

[B20] Lawson C. D. Fan C. Mitin N. Baker N. M. George S. D. Graham D. M. (2016). Rho GTPase transcriptome analysis reveals oncogenic roles for rho GTPase-activating proteins in basal-like breast cancers. *Cancer Res.* 76 3826–3837. 10.1158/0008-5472.CAN-15-2923 27216196 PMC4930678

[B21] Lee Y. C. Cai Y. Mu H. Broyde S. Amin S. Chen X. (2014). The relationships between XPC binding to conformationally diverse DNA adducts and their excision by the human NER system: is there a correlation? *DNA Repair (Amst).* 19 55–63. 10.1016/j.dnarep.2014.03.026 24784728 PMC4070384

[B22] Li Z. Musich P. R. Cartwright B. M. Wang H. Zou Y. (2013). UV-induced nuclear import of XPA is mediated by importin-α4 in an ATR-dependent manner. *PLoS One* 8:e68297. 10.1371/journal.pone.0068297 23861882 PMC3704644

[B23] Liu C. Mao Y. (2017). Formin-mediated epigenetic maintenance of centromere identity. *Small GTPases* 8 245–250. 10.1080/21541248.2016.1215658 27449713 PMC5680723

[B24] Luo J. Hosoki K. Bacsi A. Radak Z. Hegde M. L. Sur S. (2014). 8-Oxoguanine DNA glycosylase-1-mediated DNA repair is associated with Rho GTPase activation and α-smooth muscle actin polymerization. *Free Radic. Biol. Med.* 73 430–438. 10.1016/j.freeradbiomed.2014.03.030 24681335 PMC4156873

[B25] Magalhães Y. T. Farias J. O. Monteiro L. F. Forti F. L. (2018). Measuring the contributions of the Rho pathway to the DNA damage response in tumor epithelial cells. *Methods Mol. Biol.* 1821 339–355. 10.1007/978-1-4939-8612-5_23 30062423

[B26] Mamouni K. Cristini A. Guirouilh-Barbat J. Monferran S. Lemarié A. Faye J.-C. (2014). RhoB promotes γH2AX dephosphorylation and DNA double-strand break repair. *Mol. Cell. Biol.* 34 3144–3155. 10.1128/MCB.01525-13 24912678 PMC4135599

[B27] Maria Berra C. De Oliveira C. S. Machado Garcia C. C. Reily Rocha C. R. Koch Lerner L. De Andrade Lima L. C. (2013). Nucleotide excision repair activity on DNA damage induced by photoactivated methylene blue. *Free Radic. Biol. Med.* 61 343–356. 10.1016/j.freeradbiomed.2013.03.026 23567189

[B28] Mokady D. Meiri D. (2015). RhoGTPases – a novel link between cytoskeleton organization and cisplatin resistance. *Drug Resist. Updat.* 19 22–32. 10.1016/J.DRUP.2015.01.001 25660168

[B29] Mullenders L. H. F. (2018). Solar UV damage to cellular DNA: from mechanisms to biological effects. *Photochem. Photobiol. Sci.* 17 1842–1852. 10.1039/c8pp00182k 30065996

[B30] Musich P. R. Li Z. Zou Y. (2017). Xeroderma pigmentosa group a (XPA), nucleotide excision repair and regulation by ATR in response to ultraviolet irradiation. *Adv. Exp. Med. Biol.* 996 41–54. 10.1007/978-3-319-56017-5_4 29124689 PMC6597250

[B31] Oh K. S. Bustin M. Mazur S. J. Appella E. Kraemer K. H. (2011). UV-induced histone H2AX phosphorylation and DNA damage related proteins accumulate and persist in nucleotide excision repair-deficient XP-B cells. *DNA Repair (Amst).* 10 5–15. 10.1016/j.dnarep.2010.09.004 20947453 PMC3010519

[B32] Osaki J. H. Espinha G. Magalhaes Y. T. Forti F. L. (2016). Modulation of RhoA GTPase activity sensitizes human cervix carcinoma cells to γ -radiation by attenuating DNA repair pathways. *Oxid. Med. Cell. Longev.* 2016 1–11. 10.1155/2016/6012642 26649141 PMC4662998

[B33] Pfitzer L. Moser C. Gegenfurtner F. Arner A. Foerster F. Atzberger C. (2019). Targeting actin inhibits repair of doxorubicin-induced DNA damage: a novel therapeutic approach for combination therapy. *Cell Death Dis.* 10 1–14. 10.1038/s41419-019-1546-9 30944311 PMC6447524

[B34] Rastogi R. P. Richa Kumar A. Tyagi M. B. Sinha R. P. (2010). Molecular mechanisms of ultraviolet radiation-induced DNA damage and repair. *J. Nucleic Acids* 2010:592980. 10.4061/2010/592980 21209706 PMC3010660

[B35] Ray A. Blevins C. Wani G. Wani A. A. (2016). ATR- and ATM-Mediated DNA damage response is dependent on excision repair assembly during G1 but not in S phase of cell cycle. *PLoS One* 11:e0159344. 10.1371/journal.pone.0159344 27442013 PMC4956099

[B36] Rünger T. M. Kappes U. P. (2008). Mechanisms of mutation formation with long-wave ultraviolet light (UVA). *Photodermatol. Photoimmunol. Photomed.* 24 2–10. 10.1111/j.1600-0781.2008.00319.x 18201350

[B37] Russo L. C. Minaya P. Y. Silva L. E. Forti F. L. (2018). Assessing the roles of Rho GTPases in cell DNA repair by the nucleotide excision repair pathway. *Methods Mol. Biol.* 1821 319–338. 10.1007/978-1-4939-8612-5_22 30062422

[B38] Sahai E. Olson M. F. Marshall C. J. (2001). Cross-talk between Ras and Rho signalling pathways in transformation favours proliferation and increased motility. *EMBO J.* 20 755–766. 10.1093/emboj/20.4.755 11179220 PMC145410

[B39] Schuch A. P. Menck C. F. M. (2010). The genotoxic effects of DNA lesions induced by artificial UV-radiation and sunlight. *J. Photochem. Photobiol. B Biol.* 99 111–116. 10.1016/j.jphotobiol.2010.03.004 20371188

[B40] Schuch A. P. Moreno N. C. Schuch N. J. Menck C. F. M. Garcia C. C. M. (2017). Sunlight damage to cellular DNA: focus on oxidatively generated lesions. *Free Radic. Biol. Med.* 107 110–124. 10.1016/j.freeradbiomed.2017.01.029 28109890

[B41] Seifermann M. Epe B. (2017). Oxidatively generated base modifications in DNA: not only carcinogenic risk factor but also regulatory mark? *Free Radic. Biol. Med.* 107 258–265. 10.1016/j.freeradbiomed.2016.11.018 27871818

[B42] Sertic S. Pizzi S. Lazzaro F. Plevani P. Muzi-Falconi M. (2012). NER and DDR: classical music with new instruments. *Cell Cycle* 11 668–674. 10.4161/cc.11.4.19117 22373527

[B43] Spivak G. (2015). Nucleotide excision repair in humans. *DNA Repair (Amst).* 36 13–18. 10.1016/j.dnarep.2015.09.003 26388429 PMC4688078

[B44] Stevenson A. F. G. Lange C. S. (1997). Extracellular matrix (ECM) and cytoskeletal modulation of cellular radiosensitivity. *Acta Oncol. (Madr).* 36 599–606. 10.3109/02841869709001322 9408150

[B45] Toutfaire M. Bauwens E. Debacq-Chainiaux F. (2017). The impact of cellular senescence in skin ageing: a notion of mosaic and therapeutic strategies. *Biochem. Pharmacol.* 142 1–12. 10.1016/j.bcp.2017.04.011 28408343

[B46] Tseliou M. Al-Qahtani A. Alarifi S. Alkahtani S. H. Stournaras C. Sourvinos G. (2016). The role of RhoA, RhoB and RhoC GTPases in cell morphology, proliferation and migration in human cytomegalovirus (HCMV) infected glioblastoma cells. *Cell. Physiol. Biochem.* 38 94–109. 10.1159/000438612 26741994

[B47] Vogelsgesang M. Pautsch A. Aktories K. (2007). C3 exoenzymes, novel insights into structure and action of Rho-ADP-ribosylating toxins. *Naunyn Schmiedebergs Arch. Pharmacol.* 374 347–360. 10.1007/s00210-006-0113-y 17146673

[B48] Wakasugi M. Sasaki T. Matsumoto M. Nagaoka M. Inoue K. Inobe M. (2014). Nucleotide excision repair-dependent DNA double-strand break formation and ATM signaling activation in mammalian quiescent cells. *J. Biol. Chem.* 289 28730–28737. 10.1074/jbc.M114.589747 25164823 PMC4192521

[B49] Wang H. Huang Y. Shi J. Zhi Y. Yuan F. Yu J. (2019). XPC deficiency leads to centrosome amplification by inhibiting BRCA1 expression upon cisplatin-mediated DNA damage in human bladder cancer. *Cancer Lett.* 444 136–146. 10.1016/j.canlet.2018.12.004 30579971

[B50] Wheeler A. P. Ridley A. J. (2004). Why three Rho proteins? RhoA, RhoB, RhoC, and cell motility. *Exp. Cell Res.* 301 43–49. 10.1016/j.yexcr.2004.08.012 15501444

[B51] Wu X. Shell S. M. Liu Y. Zou Y. (2007). ATR-dependent checkpoint modulates XPA nuclear import in response to UV irradiation. *Oncogene* 26 757–764. 10.1038/sj.onc.1209828 16862173 PMC3106104

[B52] Zhang S. Tang Q. Xu F. Xue Y. Zhen Z. Deng Y. (2009). RhoA regulates G1-S progression of gastric cancer cells by modulation of multiple INK4 family tumor suppressors. *Mol. Cancer Res.* 7 570–580. 10.1158/1541-7786.MCR-08-0248 19372585

[B53] Zhao L. Todd Washington M. (2017). Translesion synthesis: insights into the selection and switching of DNA polymerases. *Genes (Basel)* 8:24. 10.3390/genes8010024 28075396 PMC5295019

[B54] Zheng H. Jarvis I. W. H. Bottai M. Dreij K. Stenius U. (2019). TGF beta promotes repair of bulky DNA damage through increased ERCC1/XPF and ERCC1/XPA interaction. *Carcinogenesis* 40 580–591. 10.1093/carcin/bgy156 30418489

