# Supplementary material for: RHOAming Through the Nucleotide Excision Repair Pathway as a Mechanism of Cellular Response Against the Effects of UV Radiation
Source: Front Cell Dev Biol. 2020 Aug 19;8:816. doi: 10.3389/fcell.2020.00816 (PMC7509447; doi:10.3389/fcell.2020.00816)
Supplement: Supplementary file 1 [file Data_Sheet_1.PDF]

SUPPLEMENTARY METHODS

Pull-down assays of Rho GTPase activity

The production of Rho-binding domain (RBD-GST) fusion protein was performed as previously described(Griesi-Oliveira et al., 2018). To measure the endogenous RhoA activity, *in vitro* assays of pull-down were performed following a well-established protocol formerly described(Espinha et al., 2016). Briefly, cell lysates at desired conditions were incubated with RBD-GST fusion protein that specifically bounds to RhoA-GTP (Rho active). Proteins were resolved on 13% SDS-PAGE and analyzed using a monoclonal anti-RhoA antibody (Table 1).

Analysis of actin filaments on stress fibers by immunofluorescence

The analysis of actin filaments present on stress fibers was performed as previously described(Espinha et al., 2015). Briefly, cells were fixed with 3% formaldehyde for 10 min, permeabilized with 0.5% Triton X-100 for 5 min on ice and blocked with 3% BSA/10% FBS for 30 min. F-actin was stained with anti-Phalloidin antibody conjugated with Alexa Fluor 488 (Invitrogen, 1:500 in PBS) for 2 h in a humidified dark chamber and Vectashield with DAPI (VectorLab). Images were captured by confocal microscopy using a Zeiss LSM-510 microscope.

Scratch wound healing assays

Two-dimension cell migration was measured using the wound healing assay as previously described(Ascer et al., 2015). Briefly, cells were plated at 100% confluence, subjected to radiation treatments and then the plates were diametrically scratched with a yellow pipette tip. Cell migration was evaluated by comparing the invasion of the wound area after 24h using an inverted Olympus microscope and Cell-F software (Olympus).

Autophagy analysis by checking the expression of LC3BI/II protein

The analysis of cellular autophagy was performed by the immunodetection of the LC3BI/II marker by immunofluorescence and western blotting assays. For immunofluorescence, HeLa and RhoA-N19 cells were treated with UV-radiation and collected 48 h after. The first incubation was performed with the primary antibody anti-LC3BI/II (Invitrogen, L10382, 1:500 in PBS) for 1 h at room temperature. The second incubation was made with the secondary antibody anti-rabbit Alexa 488 and anti-Phalloidin Rod (Invitrogen), both diluted 1:500 in PBS, for 1 h at room temperature. Images were visualized and captured using a Zeiss LSM-510 confocal microscope. For western blotting the same cellular conditions used for the immunofluorescence analysis was performed and total proteins resolved in a SDS-PAGE, followed by incubating the membrane with the primary antibody anti-LC3BI/II diluted 1:1000 in TBS-T, for 18 h at 4°C. Next, following the incubation with secondary antibody anti-rabbit IRDye 680RD (LI-COR Biosciences) diluted 1:15000 for 1 h at room temperature, the membrane was scanned using an Odyssey infrared imaging system (Li-Cor). As positive control for both experiments, cells were treated with 50 µM chloroquine for 16 h at 37°C.

REFERENCES

Ascer, L. G., Magalhaes, Y. T., Espinha, G., Osaki, J. H., Souza, R. C., and Forti, F. L. (2015). CDC42 Gtpase Activation Affects Hela Cell DNA Repair and Proliferation Following UV Radiation-Induced Genotoxic Stress. *J Cell Biochem* 116, 2086–2097. doi:10.1002/jcb.25166.

Espinha, G., Osaki, J. H., Costa, E. T., and Forti, F. L. (2016). Inhibition of the RhoA GTPase Activity Increases Sensitivity of Melanoma Cells to UV Radiation Effects. *Oxid Med Cell Longev* 2016, 2696952. doi:10.1155/2016/2696952.

Espinha, G., Osaki, J. H., Magalhaes, Y. T., and Forti, F. L. (2015). Rac1 GTPase-deficient HeLa cells present reduced DNA repair, proliferation, and survival under UV or gamma irradiation. *Mol Cell Biochem* 404, 281–297. doi:10.1007/s11010-015-2388-0.

Griesi-Oliveira, K., Suzuki, A. M., Alves, A. Y., Mafra, A. C. C. N., Yamamoto, G. L., Ezquina, S., et al. (2018). Actin cytoskeleton dynamics in stem cells from autistic individuals. *Sci. Rep.* 8, 11138. doi:10.1038/s41598-018-29309-6.
