# Supplementary material for: RHOAming Through the Nucleotide Excision Repair Pathway as a Mechanism of Cellular Response Against the Effects of UV Radiation
Source: Front Cell Dev Biol. 2020 Aug 19;8:816. doi: 10.3389/fcell.2020.00816 (PMC7509447; doi:10.3389/fcell.2020.00816)
Supplement: Supplementary file 2 [file Data_Sheet_2.PDF]

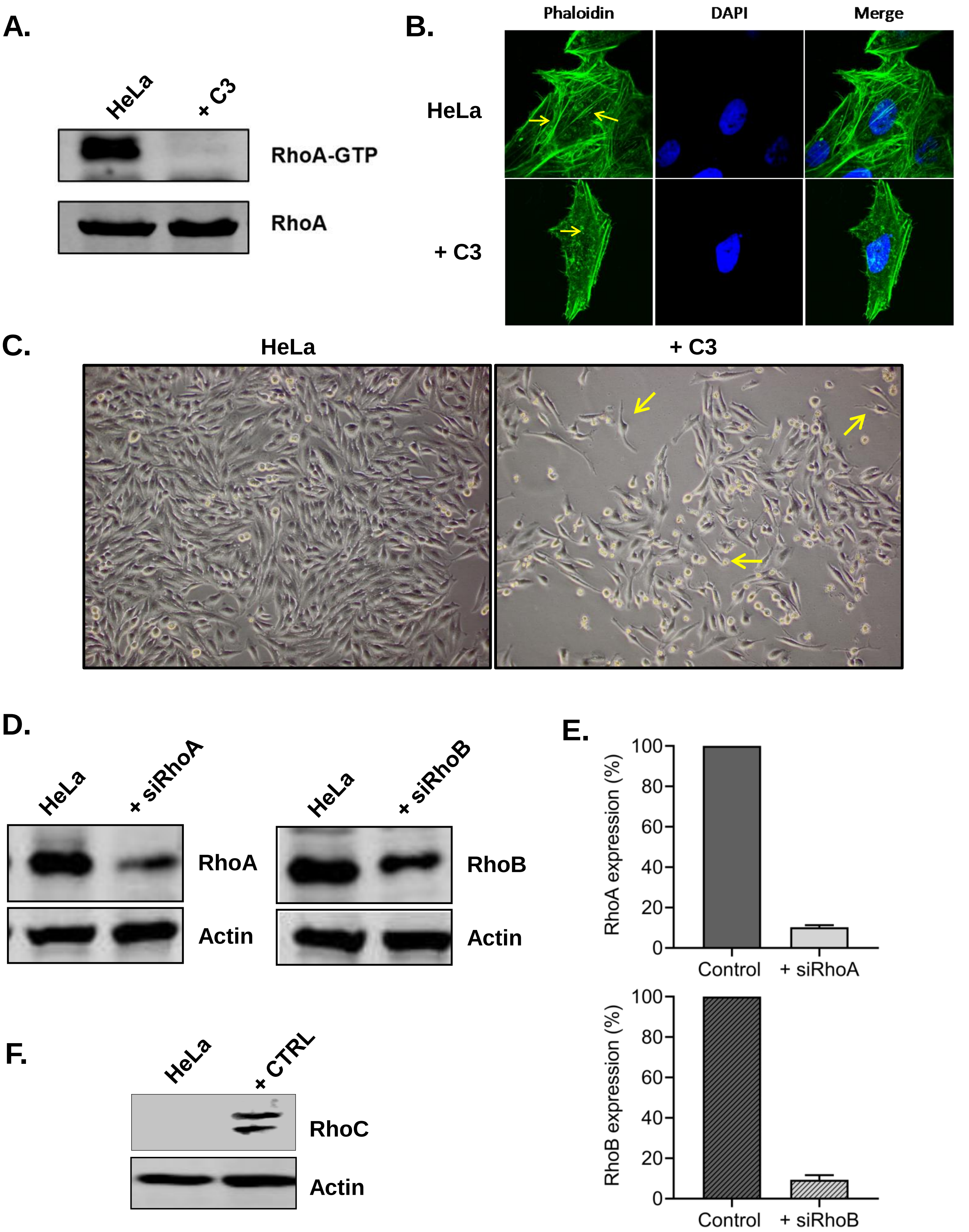

Supplementary Figure 1. Characterization of HeLa cells under different Rho *LoF* strategies. C3 toxin strongly inhibits RhoA activity as measured by pull-down assays (A), affects F-actin / stress fibers stability (B), and cell morphology (C). RhoA and RhoB knockdown by esiRNA (Sigma-Aldrich) was highly efficient by silencing approximately 90% for both genes (D, E). The RhoC knockdown was not performed because its protein expression in HeLa cells was shown to be very low and almost undetectable compared to a positive control, the U87-MG glioblastoma cell line (F).

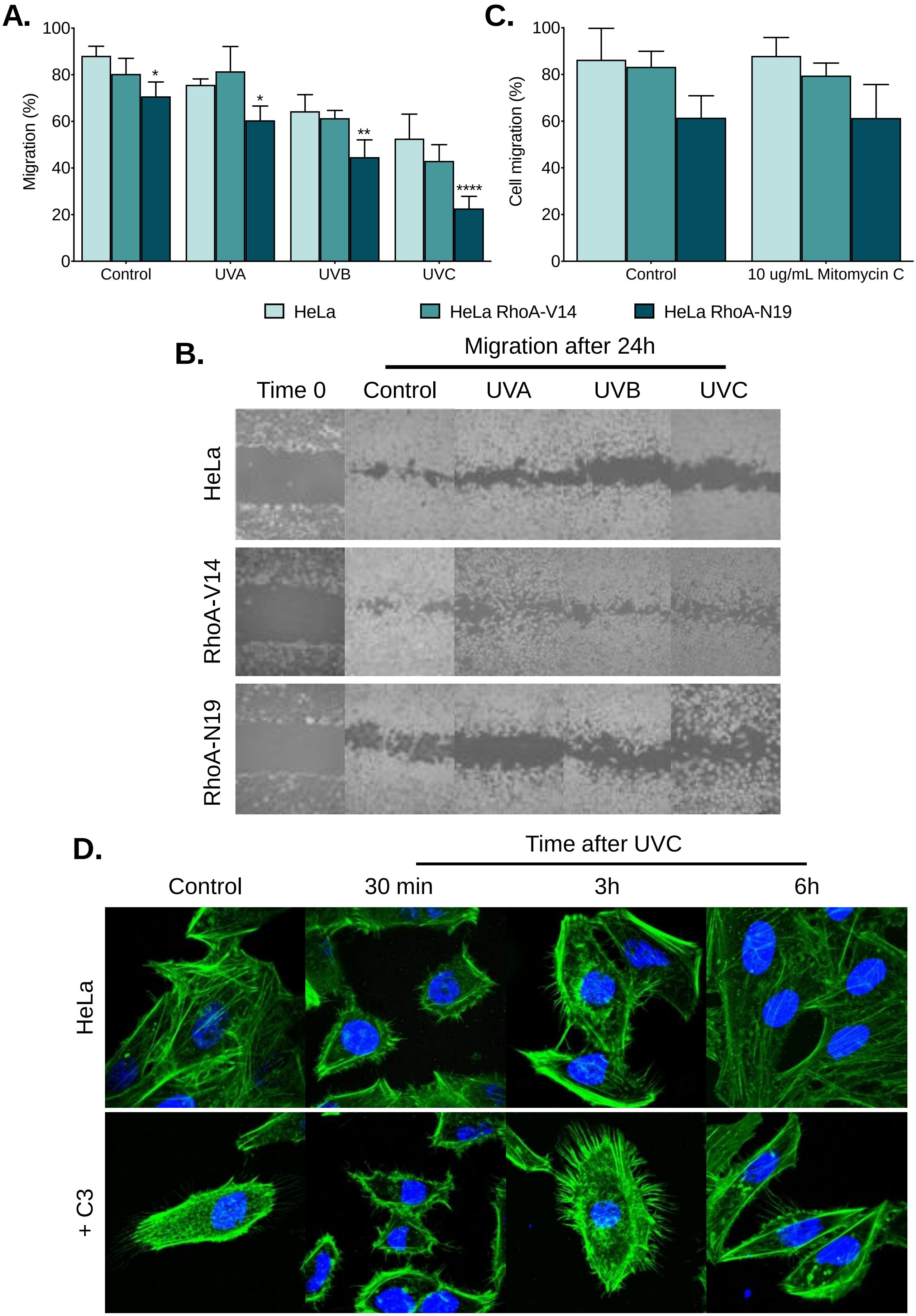

Supplementary Figure 2. Rho *LoF* affects cell migration and F-actin dynamics after UV exposure. RhoA *LoF* by ectopically expressing the RhoAN19 mutant decrease cell migration in response to different UV wavelengths (A, B). The Mitomycin C drug was used as control to eliminate possible proliferation contribution (C) along the scratch-wound healing assays. C3 toxin promotion of F-actin / stress fibers organization / stability is effectively enhanced by UV radiation stress (D).

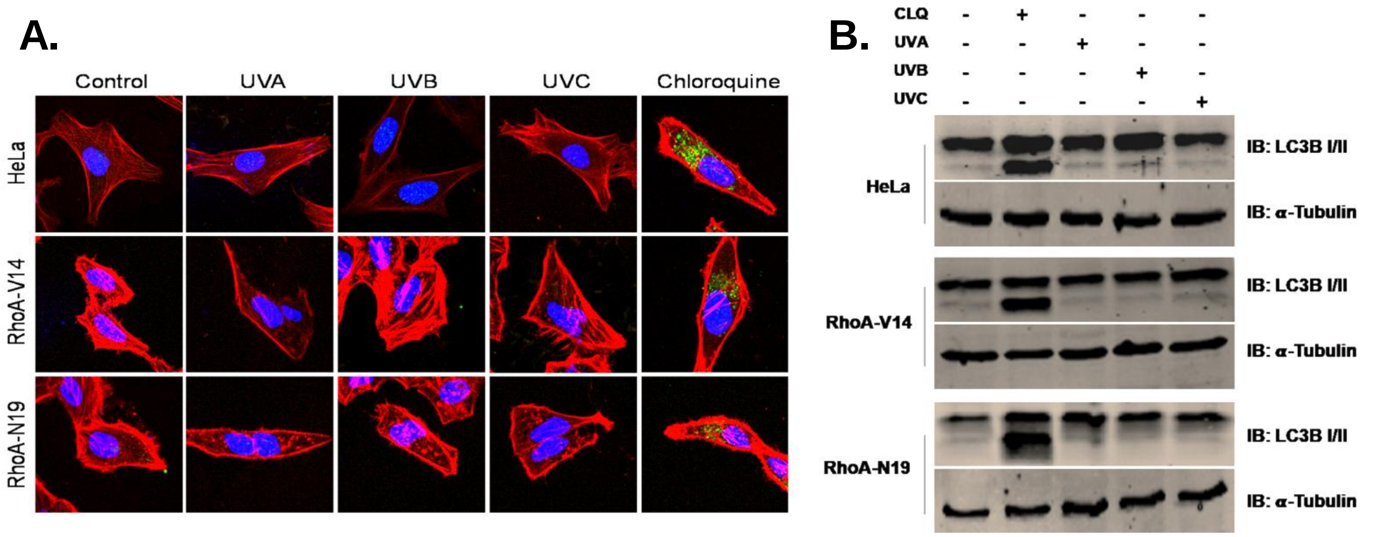

Supplementary Figure 3. Rho *LoF* does not affect autophagic cell death with or without UV stress. Immunofluorescence (A) and immunoblotting (B) for the autophagic marker LC3B I/II (showed in cytosolic green dots) show that RhoA *LoF* has no contributions to autophagic cell death in presence or absence of UV radiation. The Chloroquine (LCQ) drug (50 μM) was used as a positive control for autophagy.

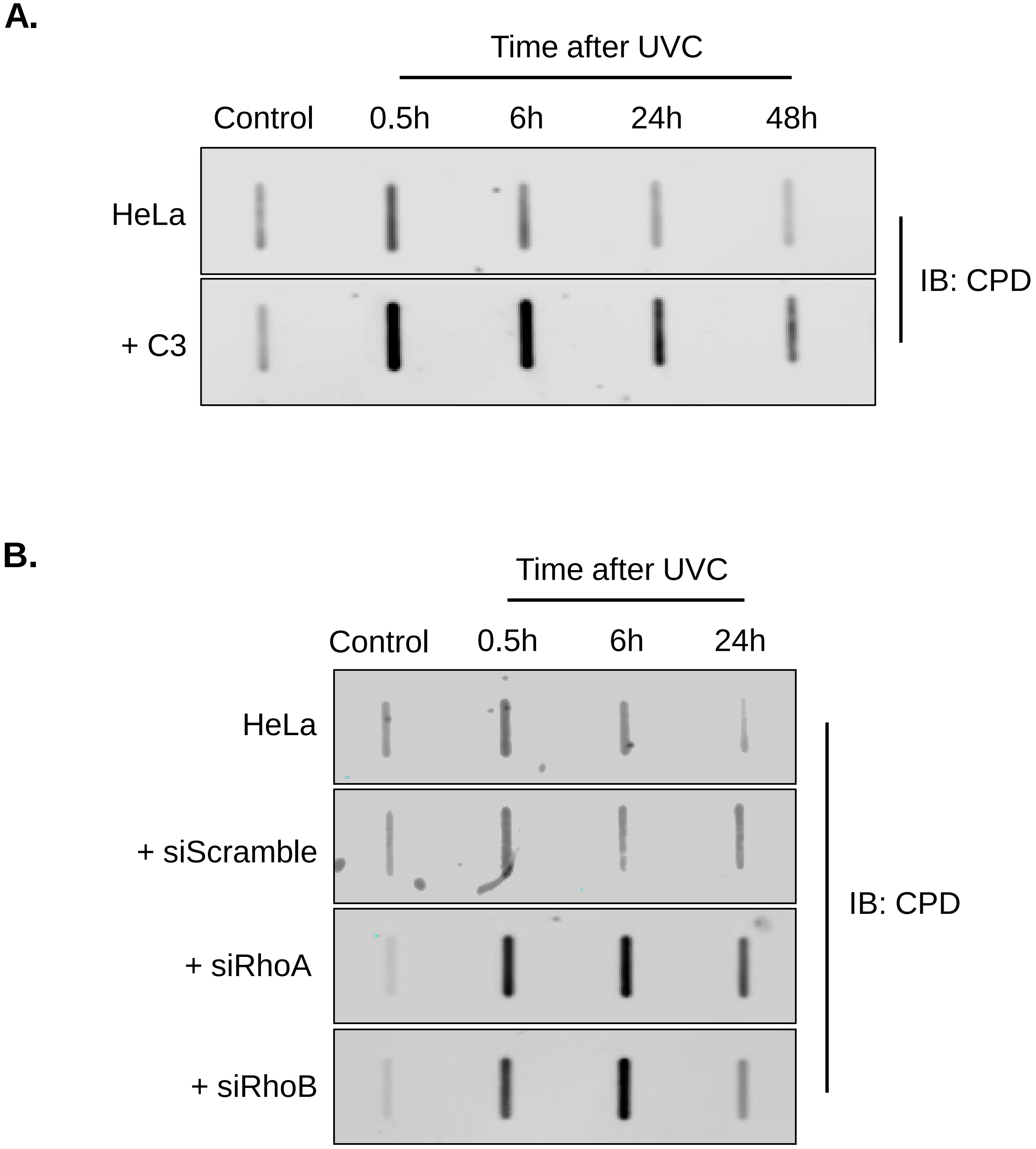

Supplementary Figure 4. CPDs detection in HeLa cells under Rho *LoF* and after UV radiation. Immuno slot-blottings for CPDs detection were performed on genomic DNA of HeLa cells where the Rho *LoF* was accomplished by either C3 inhibition (A) or knockdown of RhoA and RhoB genes (B). The blots are representative of three independent experiments and the bands quantification, normalization and statistics are showed in Figures 4B and 4C, and described in M&M section.

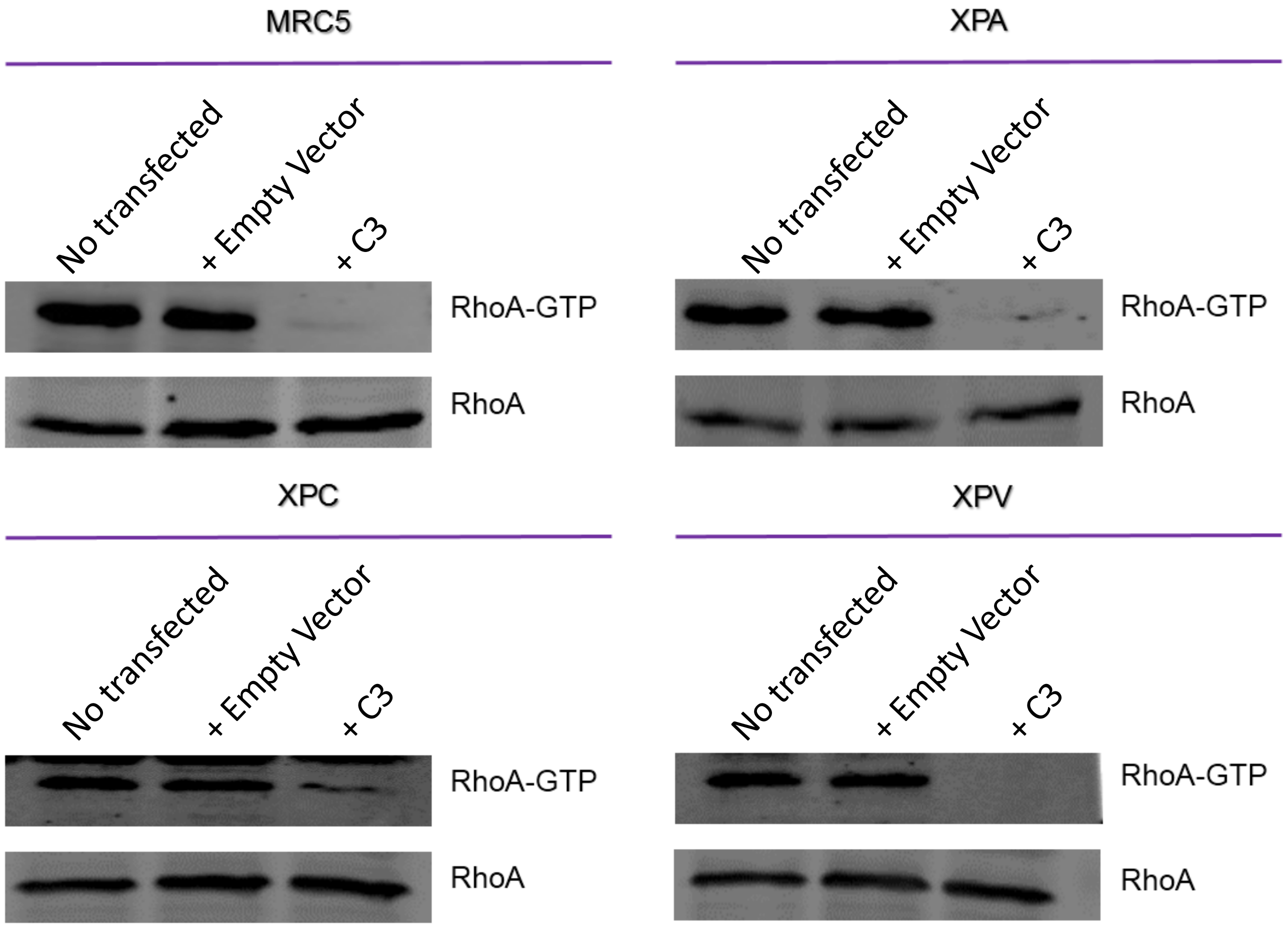

Supplementary Figure 5. Characterization Rho *LoF* using the C3 toxin method in MRC5 fibroblasts, and in NER- and TLS-deficient cell lines. Endogenous GTPasic activity of RhoA enzymes was assessed by pull-down assays, which show the potency and specificity of C3 toxin to strongly inhibit Rho activity in all the four cell lines.

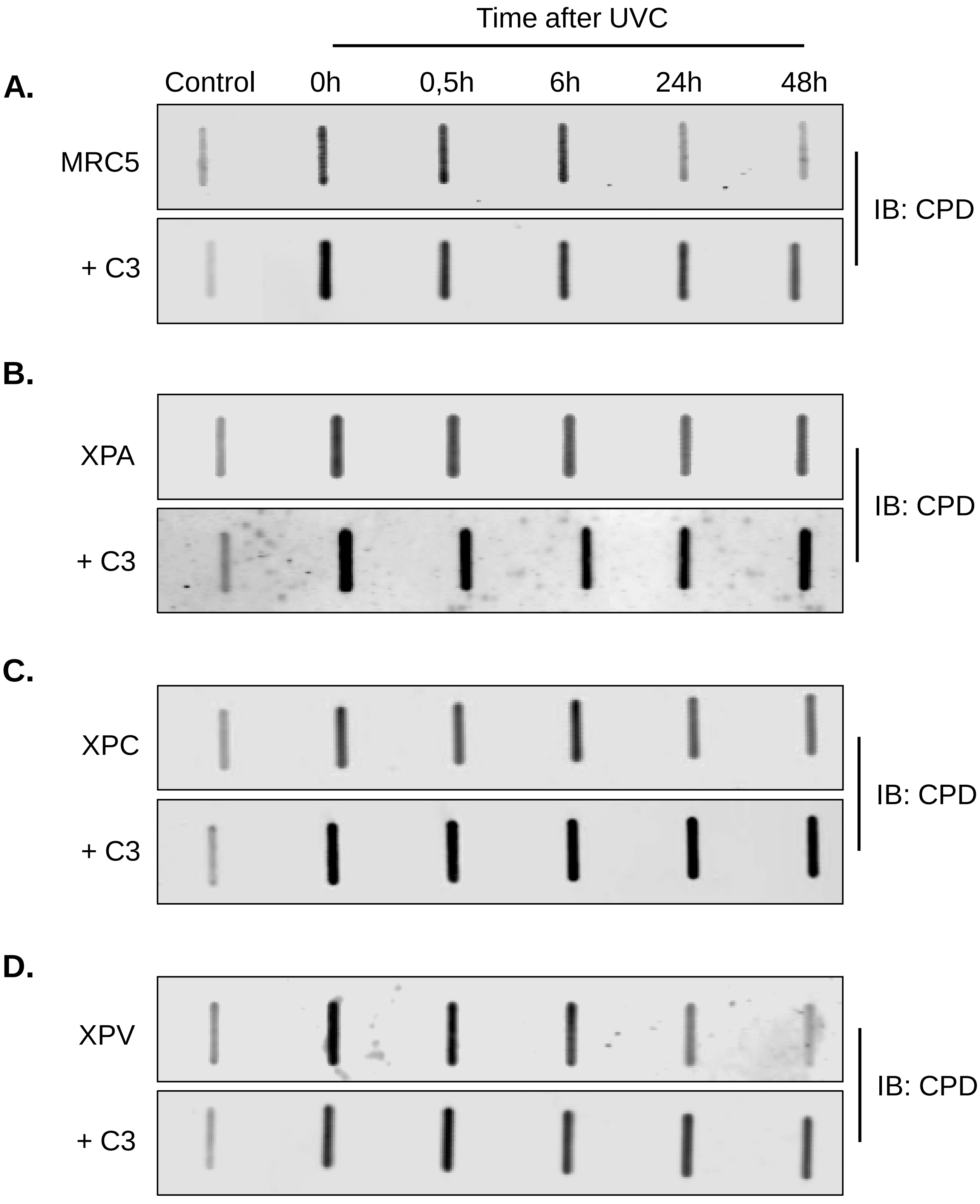

Supplementary Figure 6. CPDs detection in MRC5 fibroblasts, NER-deficient and TLS-deficient cells under Rho *LoF* and after UV stress. Immuno slot-blottings were performed for CPD detection on the genomic DNA of MRC5 fibroblasts (A), XPA-deficient (B), XPC-deficient (C), and XPV-deficient (D) cells in which the Rho *LoF* was accomplished by C3 toxin strategy previously to the UV radiation. The blots are representative of six independent experiments and the bands quantification, normalization and statistics are shown in Figures 7B and 7C and described in M&M section.

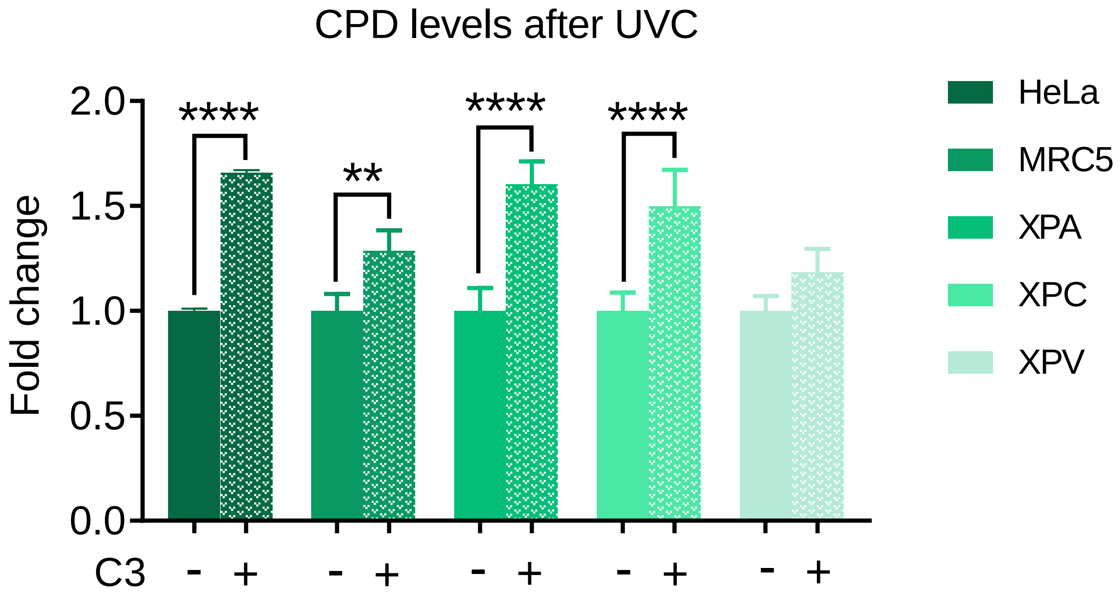

Supplementary Figure 7. Fold change variation of CPD levels in all five cell lines with or without RhoA *LoF*. The levels of CPD lesions measured in the genomic DNA of all cell lines used in this work (tumor, normal, NER-deficient and TLS-deficient) obtained from the immuno slot-blots were used to obtain the fold change caused by the RhoA *LoF per se*. A ratio between CPD bands measured immediately after exposure to UVC radiation (maximum CDP damage) and without treatment (basal control condition), correspondently without or with RhoA *LoF* by C3 toxin was obtained, and further normalized by the basal control condition without any treatment. Results show that RhoA inhibition is able to promote a differential increase in CPD levels among the cells. Graphs are average +/- SD from three independent experiments.
