# Supplementary material for: RHOAming Through the Nucleotide Excision Repair Pathway as a Mechanism of Cellular Response Against the Effects of UV Radiation
Source: Front Cell Dev Biol. 2020 Aug 19;8:816. doi: 10.3389/fcell.2020.00816 (PMC7509447; doi:10.3389/fcell.2020.00816)
Supplement: Supplementary file 3 [file Data_Sheet_3.PDF]

Supplementary Table 1. Linear regression statistics from comet assays data.

	Slope[1]	SD[1]	R square[1]	p-value[2]	Are the slopes different? [2]
			UVA		
	**Slope[1]**	**SD[1]**	**R square[1]**	**p-value[2]**	**Are the slopes different? [2]**
HeLa	-0.7344	± 0.0893	0.9576		
RhoA-N19	-0.3633	± 0.0733	0.8599	0.0132	Significant
+ C3	-0.3998	± 0.0662	0.9239		
			**UVB**		
	**Slope[1]**	**SD[1]**	**R square[1]**	**p-value[2]**	**Are the slopes different? [2]**
HeLa	-0.9767	± 0.0975	0.9616		
RhoA-N19	-0.8256	± 0.1113	0.9322	0.0295	Significant
+ C3	-0.6759	± 0.1810	0.8174		
+ siScramble	-1.244	± 0.2606	0.8506		
+ siRhoA	-0.4415	± 0.1557	0.6678	0.0135	Significant
+ siRhoB	-0.3444	± 0.1547	0.5533		
			**UVC**		
	**Slope[1]**	**SD[1]**	**R square[1]**	**p-value[2]**	**Are the slopes different? [2]**
HeLa	-0.9767	± 0.0925	0.9616		
RhoA-N19	-0.8256	± 0.1014	0.9322	0.0492	Significant
+ C3	-0.6759	± 0.1930	0.8174		
+ siScramble	-1.08	± 0.2970	0.6537		
+ siRhoA	-0.1683	± 0.1428	0.1655	0.0235	Significant
+ siRhoB	-0.3444	± 0.2166	0.2654		

[1] Results of linear regression analysis using the OTM values from the conditions of 30 min, 3h and 6h after UV radiation. The modular slope was assumed as being directly proportional to speed of repair. All statistics were obtained with GraphPad Prism 8, from three independent experiments.

[2] The statistical analysis was performed between the cells under the same UV wavelength (except for UVC, where the cells transfected with RNAi were grouped separately) using T-test and 95% confidence interval, and assumed significant when p-value <0.05.

Supplementary Table 2. Linear regression statistics from CPD lesions repair assays.

	Slope[1]	*SD*[1]	*R square*[1]	*p-value*[2]	*Are the slopes different?*[2]
HeLa	-2.059	± 0.1363	0.9580	0.0068	Very Significant
**+ *C3***	-1.597	± 0.0691	0.9816		
HeLa	-3.196	± 0.1674	0.9891	0.0014	Very Significant (Between siRhoA and other conditions)
**+ *siScramble***	-3.101	± 0.3121	0.9611		
**+ *siRhoA***	-1.507	± 0.3360	0.8341		
**+ *siRhoB***	-3.013	± 0.2650	0.9700		

[1] Results of linear regression analysis using the CPD levels from the conditions of 30 min, 6h, 24h and 48h after UV radiation. The modular slope was assumed as being directly proportional to speed of repair. All the statistics were obtained with GraphPad Prism 8, from three independent experiments.

[2] The statistical analysis was performed between HeLa and + C3 cells, and HeLa and + siRNA (siScramble, siRhoA and siRhoB) cells, using T-test and 95% confidence interval, and assumed significant when p-value <0.05.

Supplementary Table 3. Linear regression statistics from comet assays data obtained for the normal fibroblasts and XP cells.

	MRC5 fibroblasts				
	**Slope[1]**	**SD[1]**	**R square[1]**	**p-value[2]**	**Are the slopes different? [2]**
Control cells	-0.6267	± 0.0476	0.9943	0.0482	Significant
**+ *C3***	-0.4582	± 0.0451	0.9904		
	XPA-deficient cells				
	**Slope[1]**	**SD[1]**	**R square[1]**	**p-value[2]**	**Are the slopes different? [2]**
Control cells	-0.8300	± 0.0341	0.7880	0.0397	Significant
**+ *C3***	-0.1784	± 0.0397	0.0749		
	XPC-deficient cells				
	**Slope[1]**	**SD[1]**	**R square[1]**	**p-value[2]**	**Are the slopes different? [2]**
Control cells	-1.0050	± 0.2827	0.6453	0.0093	Significant
**+ *C3***	-0.8125	± 0.1742	0.7566		
	XPV-deficient cells				
	**Slope[1]**	**SD[1]**	**R square[1]**	**p-value[2]**	**Are the slopes different? [2]**
Control cells	-0.7894	± 0.0717	0.9454	0.0693	Not Significant
**+ *C3***	-0.6168	± 0.0950	0.8575		

[1] Results of linear regression analysis using the OTM values from the conditions of 30 min, 3h and 6h after UV radiation. The modular slope was assumed as being directly proportional to speed of repair. All the statistics were obtained with GraphPad Prism 8, from three independent experiments.

[2] The statistical analysis was performed between the cells under the same UV wavelength (except for UVC, where the cells transfected with RNAi were grouped separately) using T-test and 95% confidence interval, and assumed significant when p-value <0.05.

Supplementary Table 4. Linear regression statistics from CPD lesions repair assays obtained for the normal fibroblasts and XP cells.

	Slope[1]	SD[1]	R square[1]	p-value[2]	Are the slopes different? [2]
			MRC5 fibroblasts		
	**Slope[1]**	**SD[1]**	**R square[1]**	**p-value[2]**	**Are the slopes different? [2]**
Control cells	-1.8900	± 0.1925	0.9698	0.0011	Significant
**+ *C3***	-0.5219	± 0.1351	0.8326		
			XPA-deficient cells		
	**Slope[1]**	**SD[1]**	**R square[1]**	**p-value[2]**	**Are the slopes different? [2]**
Control cells	-0.0384	± 0.0596	0.1217	0.4020	Not Significant
**+ *C3***	-0.0231	± 0.0333	0.1387		
			XPC-deficient cells		
	**Slope[1]**	**SD[1]**	**R square[1]**	**p-value[2]**	**Are the slopes different? [2]**
Control cells	-0.3238	± 0.0407	0.9549	0.0004	Significant
**+ *C3***	-0.0300	± 0.0111	0.7110		
			XPV-deficient cells		
	**Slope[1]**	**SD[1]**	**R square[1]**	**p-value[2]**	**Are the slopes different? [2]**
Control cells	-1.7310	± 0.1459	0.9791	0.0106	Significant
**+ *C3***	-1.1180	± 0.0826	0.9839		

[1] Results of linear regression analysis using the CPD levels from the conditions 0h, 30 min, 6h, 24h and 48h after UV radiation. The modular slope was assumed as being directly proportional to speed of repair. All the statistics were obtained with GraphPad Prism 8, from three independent experiments.

[2] The statistical analysis was performed between HeLa and + C3 cells, and HeLa and + siRNA (siScramble, siRhoA and siRhoB) cells, using T-test and 95% confidence interval, and assumed significant when p-value <0.05.
